# Nuclear receptor REVERBα is a state-dependent regulator of liver energy metabolism

**DOI:** 10.1073/pnas.2005330117

**Published:** 2020-09-28

**Authors:** A. Louise Hunter, Charlotte E. Pelekanou, Antony Adamson, Polly Downton, Nichola J. Barron, Thomas Cornfield, Toryn M. Poolman, Neil Humphreys, Peter S. Cunningham, Leanne Hodson, Andrew S. I. Loudon, Mudassar Iqbal, David A. Bechtold, David W. Ray

**Affiliations:** ^a^Centre for Biological Timing, Faculty of Biology, Medicine and Health, University of Manchester, M13 9PT Manchester, United Kingdom;; ^b^Genome Editing Unit, Faculty of Biology, Medicine and Health, University of Manchester, M13 9PT Manchester, United Kingdom;; ^c^Oxford Centre for Diabetes, Endocrinology and Metabolism, University of Oxford, OX3 7LE Oxford, United Kingdom;; ^d^National Institute for Health Research Oxford Biomedical Research Centre, John Radcliffe Hospital, OX3 9DU Oxford, United Kingdom;; ^e^Division of Informatics, Imaging & Data Sciences, Faculty of Biology, Medicine and Health, University of Manchester, M13 9PT Manchester, United Kingdom

**Keywords:** circadian clock, energy metabolism, liver, nuclear hormone receptor, NR1D1

## Abstract

The nuclear receptor REVERBα is a core component of the circadian clock and proposed to be a dominant regulator of hepatic lipid metabolism. Using antibody-independent ChIP-sequencing of REVERBα in mouse liver, we reveal a high-confidence cistrome and define direct target genes. REVERBα-binding sites are highly enriched for consensus RORE or RevDR2 motifs and overlap with corepressor complex binding. We find no evidence for transcription factor tethering and DNA-binding domain-independent action. Moreover, hepatocyte-specific deletion of *Reverbα* drives only modest physiological and transcriptional dysregulation, with derepressed target gene enrichment limited to circadian processes. Thus, contrary to previous reports, hepatic REVERBα does not repress lipogenesis under basal conditions. REVERBα control of a more extensive transcriptional program is only revealed under conditions of metabolic perturbation (including mistimed feeding, which is a feature of the global *Reverbα*^*−/−*^ mouse). Repressive action of REVERBα in the liver therefore serves to buffer against metabolic challenge, rather than drive basal rhythmicity in metabolic activity.

## Significance

The circadian clock protein REVERBα is proposed to be a key regulator of liver metabolism. We now show that REVERBα action is critically dependent on metabolic state. Using transgenic mouse models, we show that the true role of REVERBα is to buffer against aberrant responses to metabolic perturbation, rather than confer rhythmic regulation to programs of lipid synthesis and storage, as has been thought previously. Thus, in the case of liver metabolism, the clock does not so much drive rhythmic processes, as provide protection against mistimed feeding cues. Understanding how the clock is coupled to metabolism is critical for understanding metabolic disease and the impacts of circadian disruptors such as shift work and 24-h lifestyles.

The circadian system orchestrates rhythms in behavior and physiology to anticipate and therefore optimize response to the fluctuating external environment. In line with this, clock function and cycles of energy metabolism are closely and reciprocally linked. An association between disrupted circadian rhythmicity and metabolic pathology has long been recognized (1, 2). Conversely, it is now clear that cellular and systemwide metabolic states are highly influential over the clock (3–5). In mammals, the core clock protein and nuclear receptor REVERBα is proposed to be a master regulator of energy metabolism (6, 7). Mice with global deletion of REVERBα demonstrate marked alterations in the daily balance of carbohydrate and lipid utilization and display a phenotype of substantially increased lipid accumulation and storage (8–10). This is characterized by hepatosteatosis and adipose tissue hypertrophy in normal chow-fed animals, which is exacerbated by a high-fat diet.

Expression of the *Reverbα* gene is under strong circadian control through the transactivating action of BMAL1/CLOCK heterodimers (11), and there is further posttranslational control through a circadian rhythm of REVERBα degradation (12). REVERBα therefore displays high amplitude, circadian rhythmic activity. The endogenous ligand of REVERBα is reported to be heme (13), thus the receptor is constitutively active, but may be responsive to changes in cellular redox status. In mouse liver, REVERBα shows greatest binding to the genome in the late rest phase (Zeitgeber Time [ZT] 8 to 10) (9, 14), with a nadir in the late active phase (ZT20 to 22), corresponding with protein abundance. REVERBα is a constitutive repressor and competes with activating ROR proteins at RORE sites (AGGTCA hexamer with a 5′ A/T-rich sequence) (15). In addition, REVERBα recruits the NCOR/HDAC3 corepressor complex when bound to RevDR2 motifs (paired AGGTCA hexamers with a two-nucleotide spacer) or two closely situated RORE sites (16–19) or, as has more recently been proposed, when tethered to tissue-specific transcription factors (e.g., HNF6) through mechanisms independent of direct DNA binding (20). In liver, this segregates DNA-binding domain (DBD)-dependent repression (circadian targets) from DBD-independent repression (metabolic targets). A further mechanism of REVERBα-mediated repression is the opposition of enhancer-promoter loop formation (21).

Therefore, current understanding suggests that REVERBα acts rhythmically as a major repressor of lipogenic gene expression in the liver, with peak activity toward the end of the rest phase (9). Unsurprisingly, REVERBα has been highlighted as an important potential target in tackling metabolic disease (22). However, the study of REVERBα action has been complicated by the limitations of the tools available. Widely used REVERBα ligands have been demonstrated to have nonspecific metabolic effects (23). Studies on animal models have been complicated by the pronounced behavioral and physiological impact of global deletion and by the relatively recent discovery that the widely used floxed *Reverbα*-targeting model results in a hypomorph allele, with in-frame deletion of the DNA-binding domain, rather than complete loss of receptor expression (20). Problems with antibody specificity have hampered the accurate profiling of REVERBα genome binding, so while the liver REVERBα cistrome has been mapped on multiple occasions (9, 14, 20, 21), overlap between datasets is limited.

Given that defining the actions of REVERBα is critical to understanding the complex interactions between the circadian clock and metabolism, we developed transgenic models which permit the study of REVERBα function and chromatin binding in the liver. Employing endogenous expression of HaloTag epitope-tagged REVERBα, we define a robust liver cistrome, using antibody-independent methods. Integration of this cistrome with differential gene expression in the livers of both global and hepatocyte-specific *Reverb*α knockout mice provides a striking redefinition of REVERBα function. Under homeostatic conditions, the repressive actions of REVERBα in the liver are in fact modest and do not include expected metabolic targets. However, in circumstances of wider metabolic perturbation, such as in the obese *Reverbα*^*−/−*^ mouse, or upon mistimed feeding, broader repressive effects of REVERBα become apparent. This understanding of REVERBα’s role, as an energy-state responsive regulator rather than an anticipatory driver of metabolic rhythms, challenges our current understanding of clock-metabolic coupling.

## Results

### CRISPR-Cas9 Creation of the *HaloReverbα* Mouse.

**Fig. 1.**
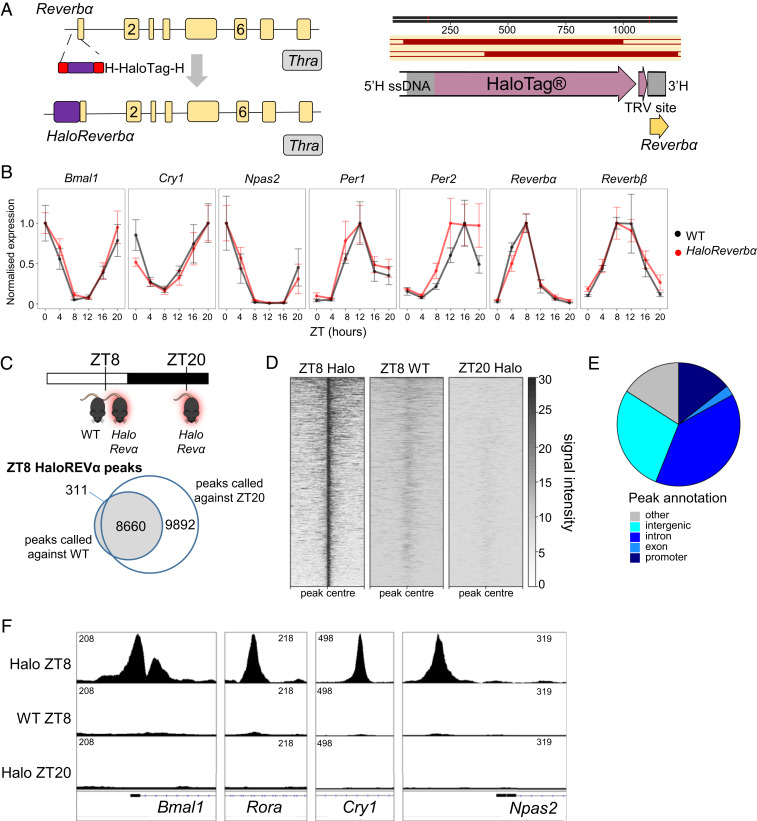
Antibody-independent ChIP-seq maps the liver REVERBα cistrome. (*A*) Targeting design for CRISPR-mediated knockin, with CRISPR target site in exon 1 of *Reverbα*, and homology flanked HaloTag long ssDNA donor *Below*. Homology-directed repair (HDR) results in the integration of HaloTag in frame with exon 1 of *Reverbα*. (*B*) Gene expression profiles of core circadian clock genes in liver tissue collected from WT (black) or *HaloReverbα* (red) mice confirming the maintenance of robust molecular rhythms with normal phase and amplitude in the *HaloReverbα* mice. Plot shows mean normalized gene expression ± SEM, *n* = 4 to 6 mice per time point. (*C*) HaloREVERBα ChIP-seq peaks were called across ZT8 *HaloReverbα* samples (*n* = 3) with ZT8 wild-type (*n* = 2) or ZT20 (*n* = 2) *HaloReverbα* samples serving as background; 8,660 peaks were common to both strategies. (*D*) Heatmap shows aggregated signal at these 8,660 sites (peak center ± 5 kbp). (*E*) Piechart of annotated peak locations shows that the majority of sites lie within a gene body or regulatory domain. (*F*) Tracks showing aggregated signal at sites associated with *Bmal1*, *Rora*, *Cry1*, and *Npas2.*

To identify a high-confidence REVERBα cistrome and circumvent potential problems of antibody specificity, we created a mouse model permitting antibody-independent ChIP-sequencing (ChIP-seq) of endogenously expressed, epitope-tagged REVERBα. We used CRISPR-Cas9 to generate mice expressing REVERBα protein (at the endogenous *Reverbα* locus) with the HaloTag protein fused to its N terminus (*HaloReverbα*) (Fig. 1*A*). We have previously used HaloTag as an effective strategy for antibody-independent chromatin immunoprecipitation in vitro (24). Insertion of the HaloTag was confirmed by sequencing. Homozygotic *HaloReverbα* mice were healthy and showed no reduction in fertility and breeding success. Under constant darkness, *HaloReverbα* mice demonstrated robust free-running rhythms and phase shift responses to both advancing and delaying light pulses were normal (*SI Appendix*, Fig. S1 *A* and *B*). However, we did observe a shortening in a period of free-running locomotor activity rhythms by ∼0.3 h in the *HaloReverbα* mice relative to wild-type (WT) controls. Given that *Reverbα*^*−/−*^ mice also display a shortening in the free-running period, albeit much more severe and with higher variability of rhythms (WT: 23.8 ± 0.1 h; *Reverbα*^*−/−*^: 23.1 ± 0.8 h; *SI Appendix*, Fig. S5*A*), it was important to confirm normal clock function in the *HaloReverbα* mice. Importantly, the addition of the HaloTag did not disturb rhythmic *Reverbα* expression or circadian clock function in the liver (Fig. 1*B*). Primary fibroblasts isolated from WT and *HaloReverbα* mice which were subsequently transduced with the PER2::LUC bioluminescence reporter (25) showed no disruption in rhythmicity or in period length (*SI Appendix*, Fig. S1*C*). No derepression of established REVERBα targets *Bmal1*, *Nfil3*, and *Npas2* was observed in either heterozygous or homozygous *HaloReverbα* mouse liver (*SI Appendix*, Fig. S1*D*), and HaloREVERBα was able to repress DR2 reporter activity in vitro (*SI Appendix*, Fig. S1*E*). This supports normal functioning of the HaloREVERBα DNA-binding domain. We have previously reported intact ligand-binding activity of the protein (26). Moreover, increased adiposity is a well-reported characteristic of *Reverbα*^*−/−*^ animals; we did not observe any adiposity phenotype in the *HaloReverbα* mice (*SI Appendix*, Fig. S1*F*). Thus, we conclude that the HaloTag only minimally affects REVERBα function, with this likely being suprachiasmatic nucleus (SCN) specific.

### HaloChIP-Seq Maps the REVERBα Cistrome in an Antibody-Independent Manner.

We therefore proceeded to HaloChIP-seq in *HaloReverbα* mouse liver using HaloLink resin pulldown. WT and *HaloReverbα* tissue was collected under light:dark conditions to ensure identical phase in sampling. We profiled the liver HaloREVERBα cistrome in homozygous *HaloReverbα* mice at ZT8 and ZT20 (peak and trough of REVERBα expression, respectively), as well as in WT littermates at ZT8. Peaks in the ZT8 *HaloReverbα* samples were called against both the ZT20 and WT datasets (to control for both the presence of the transgene and the time-of-day chromatin state, and to subtract any signal resulting from nonspecific DNA binding to the resin) (Fig. 1 *C* and *D*). We identified 8,660 peaks that were common to both analysis strategies, providing a high-confidence dataset of HaloREVERBα binding sites. We observed complete absence of HaloREVERBα chromatin binding at ZT20 (Fig. 1*D*), which confirms robust circadian rhythmicity (in terms of both expression and putative function) of the tagged protein. The 8,660 high-confidence REVERBα peaks mostly annotated to promoter, intergenic, and intronic locations (Fig. 1*E*), suggesting a role at both proximal and distal regulatory regions. As would also be expected for REVERBα, we observed peaks of strong signal on or close to core clock genes (Fig. 1*F*).

**Fig. 2.**
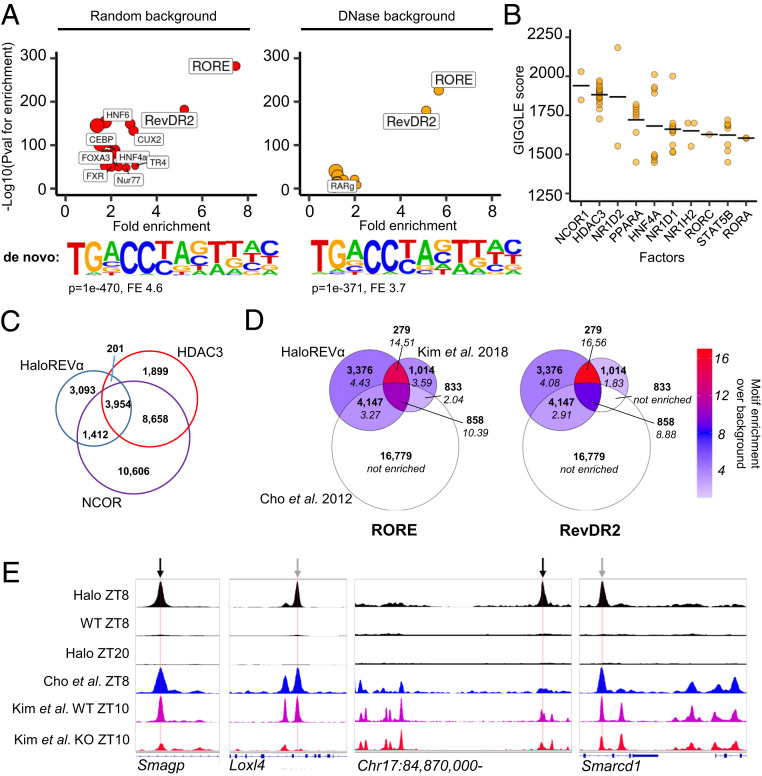
The HaloREVERBα cistrome is enriched for RORE and RevDR2 motifs, and sites of corepressor complex binding, but shows limited overlap with antibody datasets. (*A*) Motif analysis at HaloREVERBα-binding sites. Plots show enrichment of known motifs; below plot is top de novo motif identified. Analysis using random background shown on *Left*, and using a background of ZT6 to ZT10 open chromatin (DNase sites) shown on *Right*. Point size proportional to percentage of target sites containing motif. For clarity, only motifs with more than twofold enrichment are labeled; full results are in *SI Appendix*, Tables S1–S4. A RORE-like motif is the top motif identified de novo against either random or DNase background. (*B*) GIGGLE identifies transcription factors whose cistromes (top 1-k peaks, from a database of published ChIP-seq) overlap with HaloREVERBα sites. Ten factors with highest mean GIGGLE scores are plotted; full results are in *SI Appendix*, Table S5. Points indicate individual datasets, line indicates mean score. (*C*) Venn diagram shows overlap of HaloREVERBα cistrome with published HDAC3/NCOR ChIP-seq. (9). (*D*) Venn diagrams show overlap of HaloREVERBα cistrome with published antibody REVERBα ChIP-seq (14, 21). Numbers of sites are shown in bold. *Left* Venn is shaded to show enrichment of the RORE motif (over DNase background) in each group of sites; *Right* Venn is shaded to show RevDR2 enrichment. Fold-enrichment values are shown in italics. “Not enriched” = HOMER did not find that motif to be significantly enriched on standard known motif analysis. (*E*) Tracks showing aggregated ChIP-seq signal from this study and two published REVERBα studies, including REVERBα ChIP-seq performed in global REVERBα^−/−^ mice (“Kim et al. KO ZT10”; track shown in red) (21). Locations of putative RevDR2 motifs indicated with black arrows, putative RORE motifs with gray.

To further establish the validity and utility of the HaloREVERBα cistrome, we performed HOMER motif enrichment analysis of HaloREVERBα sites. Using a 200-bp window around peak centers, we found strong enrichment of the canonical motifs by which REVERBα binds the genome—RORE and RevDR2 (Fig. 2*A* and *SI Appendix*, Table S1). This is the expected pattern of DNA-binding activity of the HaloREVERBα protein and again supports its value as a model. When random genomic regions (within 50 kbp of gene transcription start sites [TSSs]; HOMER default) were used as background, characteristic hepatic transcription factor motifs (e.g., HNF6, C/EBP, and HNF4α) were also observed to be enriched in HaloREVERBα sites. These factors have been previously identified as possible tethering partners to REVERBα (20, 27). However, when regions of open chromatin identified from DNase-seq of mouse liver collected at ZT6 to ZT10 (i.e., time and tissue matched to our ChIP-seq) (28) were used as background, enrichment of these motifs was lost (Fig. 2*A* and *SI Appendix*, Table S2). This suggests that no single liver-specific transcription factor is enriched at REVERBα-binding sites, above and beyond what is normally found at sites of open chromatin in this tissue. Indeed, only RORE and RevDR2 motifs remained highly enriched at sites of REVERBα binding. De novo motif analysis yielded similar results, with the RORE motif being the most significantly enriched motif in HaloREVERBα sites, irrespective of background (Fig. 2*A* and *SI Appendix*, Tables S3 and S4). This highlights the dominance of RORE/RevDR2 motifs for determining REVERBα binding and suggests that lineage-determining factors play a permissive role by regulating chromatin accessibility, rather than directly participating in REVERBα binding to DNA by a tethering mechanism. Indeed, we phenotyped a mouse model of global REVERBα DBD mutation (*Reverbα*^*DBDm*^), which would be predicted to be spared a metabolic phenotype, given that REVERBα control of metabolism is proposed to be DBD independent (20, 27). However, we observed evidence of pronounced increase in lipid accumulation in this mouse line (increased adiposity, increased liver lipogenesis) (*SI Appendix*, Fig. S2), a phenocopy of mice globally lacking the entire REVERBα protein (*Reverbα*^*−/−*^). Together these findings suggest that REVERBα control of metabolism requires direct DNA binding mediated through RORE and RevDR2 motifs and cast doubt on the prevalence of distinct DNA-binding independent actions of REVERBα.

### The HaloREVERBα Cistrome Associates with Corepressor Complex Binding but Has Limited Overlap with Antibody-Dependent REVERBα Datasets.

We next assessed the overlap of our HaloREVERBα cistrome with those of other regulatory factors, including previously published REVERBα cistromes. We first employed GIGGLE (29), which provides an unbiased similarity score between genomic locations of interest and cistromes in the CistromeDB database. In keeping with the importance of the HDAC3/NCOR corepressor complex for functional REVERBα-mediated repression, NCOR1 and HDAC3 cistromes had the highest scoring overlap (Fig. 2*B* and *SI Appendix*, Table S5), as did REVERBα’s paralogue, REVERBβ (NR1D2), which has been reported to share the majority of its binding sites with REVERBα (30). Overlap with existing REVERBα (NR1D1) cistromes, plus those of RORα (RORA) and RORγ (RORC), gives further confidence in our HaloREVERBα cistrome. We next directly compared the HaloREVERBα cistrome with published liver cistromes for HDAC3, NCOR (9), and REVERBα (14, 21). Totals of 70% and 53% of HaloREVERBα sites showed overlap with NCOR and HDAC3 sites, respectively (Fig. 2*C*). As expected, both the HDAC3 and NCOR cistromes extend well beyond HaloREVERBα sites, reflecting that both are known to interact with other transcription factors to bring about gene repression (9, 31).

To compare the HaloREVERBα cistrome to published antibody-dependent REVERBα cistromes, we downloaded two raw datasets and called peaks as follows: liver ChIP-seq performed at ZT8 (14) with peaks called against input (resulting in 22,617 peaks); and ChIP-seq at ZT10 in WT and *Reverbα*^*−/−*^ mouse livers (21), with WT peaks called against knockout (resulting in 2,984 peaks). Examined together, these three cistromes shared 858 sites [equivalent to 9.9% of the Halo cistrome, 28.8% of Kim et al. (21) and 3.8% of Cho et al. (14)], in which 10- and 8-fold enrichment of the RORE and RevDR2 motifs was detected, respectively (Fig. 2*D*). Even stronger enrichment of the RORE and RevDR2 motifs was evident in the additional 279 sites which were common to the Halo and Kim et al. (21) datasets (14- and 16-fold, respectively). Both motifs were significantly enriched in the remaining 7,523 HaloREVERBα sites. Inspection of signal tracks from both of the antibody-dependent datasets [Cho et al. (14) and Kim et al. (21)] reveals REVERBα ChIP-seq signal at sites also detected in the *Reverbα*^*−/−*^ mouse liver (21) (examples shown in Fig. 2*E*), which suggests that these are nonspecific, false positive binding events. It is of note that other groups have found antibody-based REVERBα ChIP-seq challenging (32, 33). As with any ChIP-seq dataset, we cannot completely exclude stochastic, nonproductive interactions causing a false positive signal in the Halo experiment. Clearly, the sensitivity of the HaloREVERBα cistrome is not 100% [some RORE enrichment is seen in the 1,847 Kim et al. (21) peaks not detected by Halo]. However, taken together, motif and corepressor complex-binding analyses support superior specificity and sensitivity of the HaloREVERBα cistrome compared to antibody-dependent approaches.

### Liver-Selective Deletion of *Reverbα* Has Limited Impact on Phenotype and Transcriptome.

We wished to determine which genes are directly regulated by REVERBα, so aimed to compare the HaloREVERBα cistrome to gene targets of REVERBα in liver. To identify which genes were affected by tissue-specific REVERBα loss, we used CRISPR-Cas9 to create a mouse model with loxP sites flanking exons 2 to 6 of the *Reverbα* gene (*Reverbα*^*Flox2-6*^) (*SI Appendix*, Fig. S3*A*). We crossed this line with the tamoxifen-inducible *Alb*^*CreERT2*^ driver line (34) to produce mice in which *Reverbα* could be selectively deleted in hepatocytes in adult animals (*Reverbα*^*Flox2-6*^*Alb*^*CreERT2*^) (*SI Appendix*, Fig. S3 *B* and *C*).

**Fig. 3.**
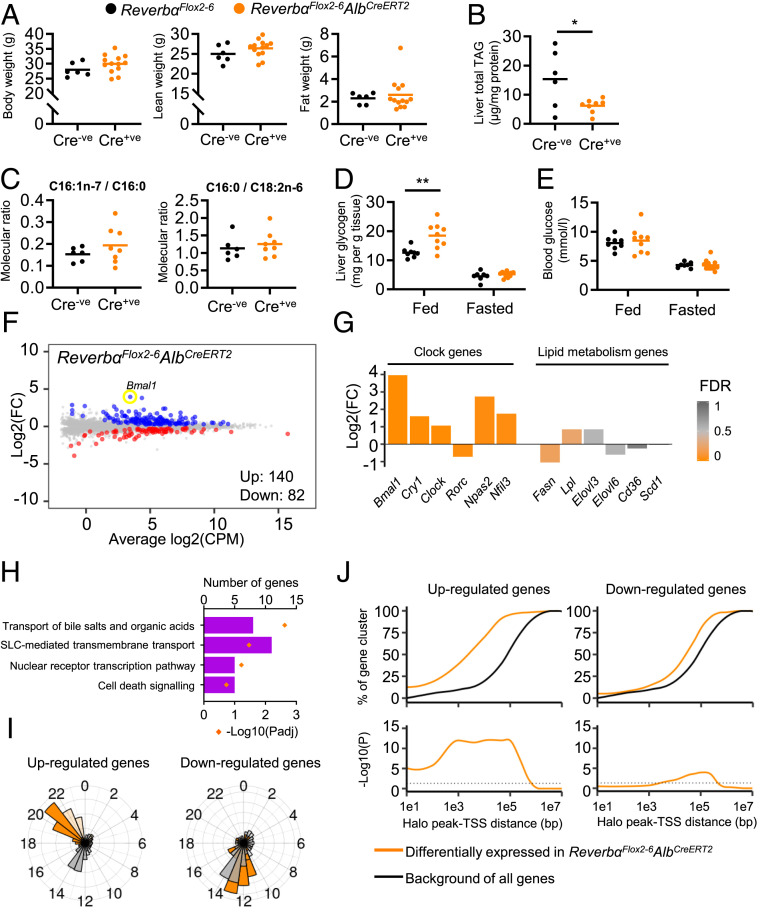
Liver-targeted *Reverbα* deletion and HaloREVERBα ChIP-seq define direct REVERBα targets. (*A*) No genotype differences in body weight, lean, and fat mass were observed between *Reverbα*^*Flox2-6*^*Alb*^*CreERT2*^ mice and *Reverbα*^*Flox2-6*^ littermate controls (12- to 14-wk-old males, *n* = 6 to 13 per group, 15 d after the first tamoxifen injection [10 d after the last]). (*B* and *C*) *Reverbα*^*Flox2-6*^*Alb*^*CreERT2*^ mice do not accumulate liver lipid. Quantification of total liver triacylglyceride (TAG) (*B*) and fatty acid composition analysis (*C*) show diminished TAG levels in *Reverbα*^*Flox2-6*^*Alb*^*CreERT2*^ liver (compared to controls) and no differences in fatty acid desaturation (reflected by C16:1n-7/C16:0 ratio) or de novo lipogenesis (reflected by C16:0/C18:2n ratio). (*n* = 6 to 8 per group, 12- to 14-wk-old males and females, 15 d after the first tamoxifen injection). (*D* and *E*) Liver-specific deletion of REVERBα has some effect on carbohydrate metabolism, as shown by increased liver glycogen in the fed state in *Reverbα*^*Flox2-6*^*Alb*^*CreERT2*^ mice (*D*), but no differences in blood glucose levels (*E*) (*n* = 7 to 12 per group, 12- to 14-wk-old males and females, 15 d after the first tamoxifen injection). (*F*) Mean difference (MD) plot of liver RNA-seq from *Reverbα*^*Flox2-6*^*Alb*^*CreERT2*^ male mice (relative to littermate controls); genes with significant differential expression (FDR < 0.05) highlighted in color (up-regulated in blue, down-regulated in red). (*G*) Circadian clock, but not lipid metabolism genes are differentially expressed (DE) in the livers of *Reverbα*^*Flox2-6*^*Alb*^*CreERT2*^ mice compared to controls (fold change [FC] and FDR of selected clock and lipid metabolism genes from *F*). (*H*) Reactome pathway analysis of significantly DE genes does not show enrichment of lipid metabolism pathways. Four pathways with lowest *P*adj values are shown. Full results are in *SI Appendix*, Table S6. (*I*) Phase plots of rhythmic, differentially expressed genes identified in *Reverbα*^*Flox2-6*^*Alb*^*CreERT2*^ livers (orange), with all rhythmic genes in liver shown in gray. Gene rhythmicity and peak phase (acrophase) were taken from ref. 32. Introns are in darker bars, exons in lighter bars. (*J*) Proximity of differentially expressed genes to HaloREVERBα peaks. (*J*, *Top*) Proportion of each gene set lying within a given distance of peaks. (*J*, *Bottom*) Significance of the enrichment of each gene set compared to all genes in the genome at each distance. Individual data points are shown, line at mean, *P < 0.05, **P < 0.01, unpaired *t* test (*A*–*C*). Two-way ANOVA with Tukey’s multiple comparisons tests (*D* and *E*).

Contrary to expectation (8, 10, 20), there was no discernible impact of liver-specific deletion of *Reverbα* on body weight or overall body composition (Fig. 3*A* and *SI Appendix*, Fig. S3*D*), nor an impact on liver triacylglyceride (TAG) content (Fig. 3*B*) at either 15 or 25 d after the start of tamoxifen treatment. In fact, lower total TAG levels were observed in *Reverbα*^*Flox2-6*^*Alb*^*CreERT2*^ mice compared to littermate controls (Fig. 3*B*). These findings are in marked contrast to the phenotype of global REVERBα knockout mice (8, 10, 20). Similarly, loss of REVERBα did not cause a derepression of de novo lipogenesis (Fig. 3*C* and *SI Appendix*, Fig. S3*E*). Thus, the development of hepatosteatosis reported in liver-specific deletion of HDAC3 (9) must stem from HDAC3 normally interacting with additional transcription factors (Fig. 2*D*). We did observe an increase in liver glycogen stores in the fed state in *Reverbα*^*Flox2-6*^*Alb*^*CreERT2*^ mice (Fig. 3*D*), as has been reported in global *Reverbα*^*−/−*^ mice (8). There was no concurrent impact on circulating blood glucose concentration (Fig. 3*E*), suggesting that REVERBα loss does not impinge on glycogen breakdown.

These data are not consistent with REVERBα functioning as a major regulator of lipid metabolism (as it is currently considered), nor with the extensive cistrome defined above. Therefore, we performed RNA-seq in livers from tamoxifen-treated *Reverbα*^*Flox2-6*^*Alb*^*CreERT2*^ mice and *Reverbα*^*Flox2-6*^ littermate controls (*n* = 7 to 8 per group, age-matched males). In keeping with the minor phenotypic effects seen, transcriptional reprogramming was modest. Only 222 genes showed significant differential expression (Fig. 3*F*), with the majority of these being derepressed (up-regulated). As expected, circadian clock gene expression was significantly altered in *Reverbα*^*Flox2-6*^*Alb*^*CreERT2*^ liver (Fig. 3*G*), with *Bmal1* showing the greatest derepression. However, no dysregulation of lipogenic genes or enrichment of lipid metabolism pathways within the differentially expressed (DE) genes was detected (Fig. 3 *G* and *H* and *SI Appendix*, Table S6). Comparison of the RNA-seq gene set with a published mouse liver circadian transcriptome (35) showed that 55% of genes up-regulated in *Reverbα*^*Flox2-6*^*Alb*^*CreERT2*^ are robustly rhythmic under normal conditions. Moreover, the acrophases of these genes cluster tightly around Circadian Time (CT) 20 to 22, the nadir of REVERBα recruitment to the genome (Fig. 3*I*). This strongly suggests that these genes are the obligate targets of REVERBα repressive activity. A total of 41% of down-regulated genes are normally rhythmic, but the acrophases of these genes clustered around CT14, suggesting that these genes are not direct REVERBα targets.

### HaloREVERBα-Binding Sites Associate with REVERBα-Regulated Genes.

We next examined the relationship between genes differentially expressed in the *Reverbα*^*Flox2-6*^*Alb*^*CreERT2*^ livers with the HaloREVERBα cistrome. Using a hypergeometric test (36) with all genes in the genome as background, we found statistically significant enrichment of genes up-regulated in *Reverbα*^*Flox2-6*^*Alb*^*CreERT2*^ livers at peak TSS distances of 100 bp to 100 kbp (Fig. 3*J*). We observed close proximity between genes up-regulated in *Reverbα*^*Flox2-6*^*Alb*^*CreERT2*^ liver and HaloREVERBα peaks, with 55% of these genes lying within 5 kbp of a peak (compared to 15% of all genes), and 87% lying within 50 kbp (compared to 40% of all genes). Only minimal enrichment was observed for down-regulated genes, further suggesting that these may be regulated indirectly, consistent with REVERBα having purely repressive action.

This consistency between the transcriptional changes observed with liver REVERBα targeting and the HaloREVERBα cistrome provides powerful support for the validity of the two models. Furthermore, it supports the notion that there are a small number of genes, close to REVERBα-binding sites, which are direct REVERBα-repressed targets in liver under a basal state. However, it does not explain why the mapped HaloREVERBα cistrome is much broader (>8,000 sites) than would be required to regulate the 140 genes derepressed in response to liver-specific deletion of *Reverbα*.

### Global *Reverbα* Deletion Rewires the Liver Transcriptome and Up-Regulates Metabolic Pathways.

**Fig. 4.**
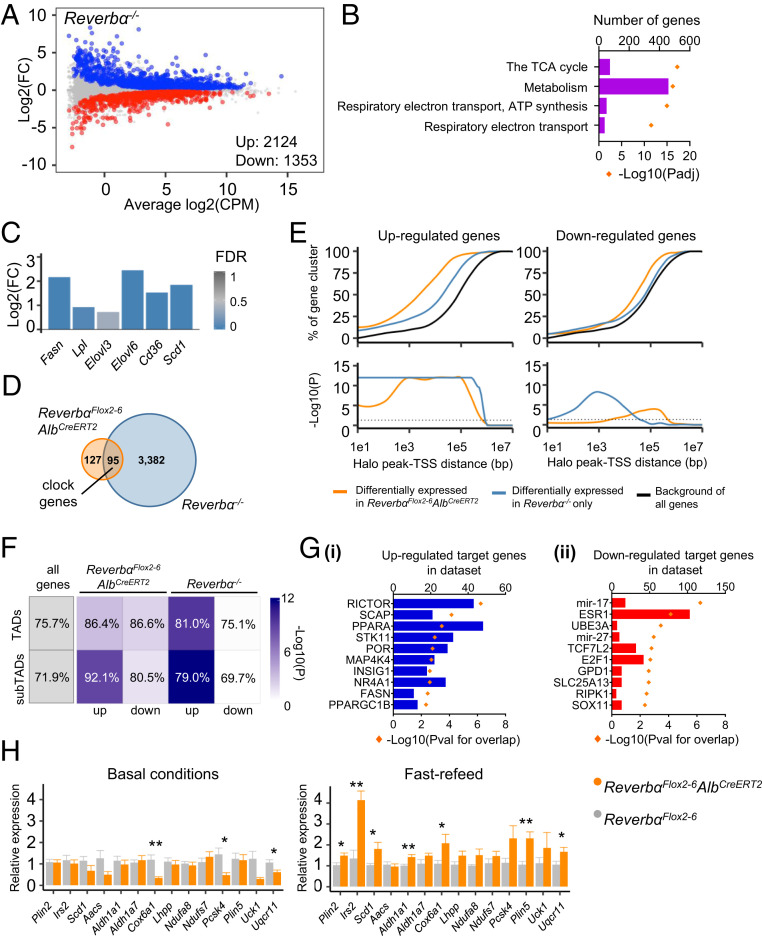
The broader repressive effects of REVERBα become evident under conditions of metabolic perturbation. (*A*) MD plot of liver RNA-seq from *Reverbα*^*−/−*^ male mice (relative to littermate controls); genes with significant differential expression (FDR < 0.05) highlighted in color (up-regulated in blue, down-regulated in red). (*B*) Reactome pathway analysis of significantly DE genes shows enrichment of metabolic pathways. Four pathways with lowest *P*adj are shown. Full results are in *SI Appendix*, Table S7. (*C*) Lipid metabolism genes are differentially expressed in the livers of *Reverbα*^*−/−*^ mice compared to controls (fold change [FC] and FDR of selected clock and lipid metabolism genes from *A*). (*D*) Overlap of significantly DE genes in each mouse model. (*E*) Proximity of DE genes to HaloREVERBα peaks in both liver-specific and global deletion of REVERBα. (*E*, *Top*) Proportion of each gene set lying within a given distance of peaks. (*E*, *Bottom*) Significance of the enrichment of each gene set compared to all genes in the genome at each distance. (*F*) Cistrome-transcriptome associations respect TAD/subTAD boundaries. Table lists percentage of DE genes from each RNA-seq cluster which are found within the same TAD/subTAD as HaloREVERBα sites. Percentage of all genes is shown for comparison. Depth of shading indicates significance of enrichment (compared to all genes). (*G*) IPA identifies regulators putatively upstream of genes which are (*i*) only up-regulated (derepressed) or (*ii*) only down-regulated in *Reverbα*^*−/−*^ livers, and which are within 100 kbp of HaloREVERBα sites. (*H*) Mistimed feeding causes significant increase in expression in *Reverbα*^*Flox2-6*^*Alb*^*CreERT2*^ liver (*Right*) at HaloREVERBα-associated genes, which are not derepressed under basal conditions (*Left*). *P < 0.05, **P < 0.01 Welch’s *t* tests, *n* = 8 to 14 per group.

The global *Reverbα*^*−/−*^ mouse exhibits a well-documented abnormal metabolic phenotype (8, 10, 20), and we hypothesized that gene expression in the livers of these mice would be similarly disturbed. Indeed, gross reprogramming of the liver transcriptome in *Reverbα*^*−/−*^ mice was evident, with ∼15% of all genes differentially expressed compared to WT littermates (Fig. 4*A*). Pathway analyses of these DE genes demonstrated pronounced enrichment of metabolic pathways (Fig. 4*B*). Up-regulated genes included important genes of lipid metabolism (Fig. 4*C*), many of which have been previously defined as targets of REVERBα transcriptional control.

Clearly, a large number of genes are deregulated in the *Reverbα*^*−/−*^ model which were not affected in *Reverbα*^*Flox2-6*^*Alb*^*CreERT2*^ mice (Fig. 4*D*). Common DE genes between the two models include the core clock genes (*Bmal1*, *Nfil3*, *Npas2*, and *Cry1*), and these are presumptive direct targets of REVERBα in the liver. As REVERB exists as two paralogues, α and β, the role of REVERBβ in the liver is of interest. However, we find a similarly small effect on gene expression (in comparison to the global *Reverbα*^*−/−*^) in a recent *Reverbα/β* liver-specific double knockout (37), and derepression of lipogenic pathways is again not observed (*SI Appendix*, Fig. S4 *A*–*C*). Thus, REVERBβ is highly unlikely to be the explanation for the divergence between our two models. Instead, the question is whether the widespread transcriptomic effects evident in *Reverbα*^*−/−*^ liver solely result from the loss of REVERBα action in this tissue, or instead are the consequence of the abnormal metabolic phenotype of these animals, acting on a REVERBα-deficient liver.

### Metabolic Perturbation Unmasks Broader REVERBα Action.

Therefore, we sought an association between DE genes identified in the *Reverbα*^*−/−*^ liver with the HaloREVERBα cistrome. Importantly, our peaks-genes analysis revealed a significant enrichment of up-regulated genes relative to HaloREVERBα sites, albeit at greater average distances than observed for genes up-regulated in *Reverbα*^*Flox2-6*^*Alb*^*CreERT2*^ liver (Fig. 4*E*). This association was much stronger than that observed for down-regulated genes. Furthermore, when we mapped DE genes and HaloREVERBα peaks to defined mouse liver topologically associating domains (TADs) and subdomains (subTADs) (21), we saw strong enrichment of REVERBα-repressed genes (from both models) within domains containing HaloREVERBα sites (Fig. 4*F*), showing that these associations respect TAD and subTAD boundaries.

These results strongly suggest that the metabolic genes derepressed in *Reverbα*^*−/−*^ liver are indeed under REVERBα control. Given the distinct metabolic phenotypes of the *Reverbα*^*−/−*^ and *Reverbα*^*Flox2-6*^*Alb*^*CreERT2*^ models, we hypothesized that wider REVERBα repression may only be unmasked under circumstances of secondary metabolic perturbation. In support of this, putative upstream regulators of genes up-regulated in *Reverbα*^*−/−*^ livers (identified by IPA upstream regulator analysis) included numerous regulators of energy metabolism such as RICTOR, SCAP (SREBP cleavage protein), and PPARα (Fig. 4*G*). By contrast, metabolic regulators were not identified by analysis of down-regulated genes.

One candidate form of metabolic perturbation is disordered feeding behavior. Both mice with brain-targeted *Reverbα* deletion and global *Reverbα*^*−/−*^ mice exhibit abnormal rhythms of feeding activity (increased daytime feeding) (38). Indeed, on closely tracking the food intake of *Reverbα*^*−/−*^ mice and comparing it to WT controls, we observed far greater variability over the course of the day, while total intake remained comparable (*SI Appendix*, Fig. S5*B*). We therefore tested the possibility that mistimed feeding may unmask additional REVERBα action in the liver by challenging tamoxifen-treated *Reverbα*^*Flox2-6*^ and *Reverbα*^*Flox2-*6^Alb^CreERT2^ mice with acute rest phase refeeding after a 24-h fast. A set of putative target genes was chosen based on the presence of a proximal HaloREVERBα ChIP peak, and significant up-regulation in the global *Reverbα*^*−/−*^, but not liver-targeted, mice. As predicted, we now observed a significantly higher expression of many of these target genes (including *Scd1* and *Irs2*) in the liver of refed *Reverbα*^*Flox2-*6^Alb^CreERT2^ mice compared to refed *Reverbα*^*Flox2-6*^ controls (Fig. 4*H*).

Taken together, our data suggest a model whereby REVERBα constitutively represses proximal target genes which, in liver, are chiefly limited to circadian clock genes. REVERBα also serves to limit the response to lipogenic cues at the wrong time of day; thus the increased daytime feeding exhibited by *Reverbα*^*−/−*^ animals, or the mistimed refeeding of *Reverbα*^*Flox2-*6^Alb^CreERT2^ mice, reveals a broader scope of REVERBα influence.

## Discussion

The circadian clock is a major regulator of energy metabolism, with REVERBα clearly serving as an influential link between the clock and metabolic state. In mice, numerous studies have demonstrated profound and pervasive effects of global REVERBα deletion, which range from enhanced adipose tissue deposition, altered bile acid, and lipid metabolism in liver, impaired oxidative capacity in muscle, through to altered feeding and adaptive behaviors (8–10, 14, 39–42).

To understand REVERBα action, we mapped a robust liver REVERBα cistrome using HaloTag technology, representing in vivo use of antibody-independent ChIP-sequencing. We did observe a shortened period in *HaloReverbα* mice under free-running conditions, but saw normal rhythmicity of liver clock gene expression and expected repression of REVERBα target genes, supporting normal functioning of the HaloREVERBα protein in the liver. On performing HaloREVERBα ChIP-seq, we found appropriate signal enrichment at clock gene loci, evidence of corepressor complex recruitment to HaloREVERBα sites and sequence motif analyses which demonstrated a high enrichment of classic RORE/RevDR2 sequences. Interestingly, enrichment for other transcription factor motifs was relatively weak, and no significant enrichment of HNF6, C/EBP, HNF4α motifs was observed beyond what would be seen at any sites of open chromatin in the liver. Therefore, we find no evidence for REVERBα-transcription factor tethering as previously proposed (20, 27). To test this we generated a global DNA-binding domain mutant animal (*Reverbα*^*DBDm*^) and indeed found that this was not spared the adiposity and liver lipid accumulation seen with global REVERBα deletion (*Reverbα*^*−/−*^).

We characterized REVERBα action in the liver by creating a mouse model permitting hepatocyte-selective *Reverbα* deletion in adulthood. Our studies revealed a relatively limited impact of REVERBα when deleted in this manner. Mice do not display enhanced lipid accumulation in the liver, nor are programs of lipogenic genes derepressed. This is in contrast to the significant phenotypic disturbance and transcriptional dysregulation observed in *Reverbα*^*−/−*^ livers. Although REVERB exists as two paralogues (α and β), in most studies REVERBα is dominant, with higher mRNA translation efficiency as the mechanism (43). Deletion of *Reverbß* alone, studied either in liver or in bronchial epithelium, does not result in obvious phenotypic abnormalities (26, 30), and analysis of data from a liver-specific double *Reverbα/β* knockout (37) confirms that the minimal phenotype seen in the liver-specific REVERBα null animal does not result from REVERBβ compensation.

Significantly, up-regulated genes identified in both liver-targeted and systemic deletions of REVERBα showed a pronounced association with the HaloREVERBα cistrome, based on proximity enrichment and clustering within TAD/subTAD chromatin regions. Thus, in intact mice, DNA-bound REVERBα must contribute to the regulation of metabolic genes, but this regulation is dependent on metabolic context. This finding echoes work suggesting that polarization signals determine the impact of *Reverbα* deletion in macrophages (32). Pathway analyses identified influential energy state-responsive factors (e.g., RICTOR, SCAP, and PPARα) as putative upstream regulators of those genes derepressed only in the context of global *Reverbα*^*−/−*^ mice. Numerous studies have demonstrated that nutritional signals also shape the chromatin landscape of mouse liver (4, 44–46), while REVERBα itself regulates higher-order chromatin structure (21). We propose that daytime recruitment of REVERBα to the genome serves to repress activity at enhancers sensitive to feeding state (e.g., through HDAC3 recruitment and regulation of enhancer-promoter loop formation), imposing a check on lipogenic cues arriving at the wrong time of day. Due to this metabolic state-dependent role, pronounced dysregulation of gene expression and metabolic phenotype is not observed in the basal state in liver-specific *Reverbα* deletion. However, as we demonstrate, challenging the liver-selective *Reverbα* knockout mice with mistimed feeding revealed an additional contingent of REVERBα-directed metabolic genes. This fits with our proposal that disordered feeding behavior in *Reverbα*^*−/−*^ mice, resulting from loss of *Reverbα* in the brain, is responsible for the up-regulation of lipogenic processes observed in the livers of these animals. Interestingly, recent reports have observed the action of other nuclear receptors to be critically dependent on metabolic context, with liver-targeted deletion of GR and RORα/γ both having diet-dependent effects (46, 47).

We therefore propose a paradigm for REVERBα action. In the basal state, REVERBα exerts repressive control over a select program of genes, which are consequently rhythmic and in antiphase to REVERBα expression. There is a far wider program of genes over which REVERBα can exert repressive control, but this is only unmasked by metabolic perturbation, for example feed/fast disruption. This advocates a major shift in our understanding of how REVERBα (and likely the rest of the circadian clock) directs gene expression across time and in response to challenge. A growing number of studies detail marked changes in rhythmic gene expression (for example in the liver) under altered metabolic, inflammatory, or pathological states (3, 5, 48). In line with these findings, our data support a model in which peripheral clock activity does not drive transcriptional rhythms in anticipation of our stereotypical daily behavioral cycles. Rather, the clock shapes tissue responses to changing systemic cues and physiological state according to time of day.

Underlining its importance, REVERBα expression exhibits a pronounced and high-amplitude rhythm in virtually all tissues, including liver, adipose, and muscle tissue. REVERBα-dependent corepressor complex recruitment and opposition of chromatin loop formation may therefore act in counterbalance to lipogenic factors arriving at the wrong time of day. In wild-type mice, ∼30% of daily food intake occurs during their normal resting period, thus REVERBα may serve to hold lipogenic responses in check following daytime bouts of feeding. In modern human society, shift work and social jetlag drive desynchrony between internal clock cycles and the external environment, including food availability, and are associated with metabolic disease (49, 50). Thus this interpretation of REVERBα’s role has important implications for time-disordered nutrition and human metabolic health.

## Methods

### Animals.

Procedures were approved by the University of Manchester Animal Welfare and Ethical Review Body and carried out under project license 70 8558 (Dr. David A. Bechtold) according to the UK Animals (Scientific Procedures) Act 1986. Animals had free access to standard laboratory chow and water, unless otherwise stated, and were group housed on 12 h:12 h light:dark cycles. Male mice (*Mus musculus*) were used, unless otherwise stated, with all RNA-seq studies being conducted on 12- to 14-wk-old weight-matched males. Experimental design and group sizes were based on previous experience and appropriate power analyses.

#### Reverbα^−/−^.

*Reverbα*^*−/−*^ mice have exons 2 to 5 of the *Reverbα* gene replaced by an in-frame LacZ allele (11). These mice were originally created by Ueli Schibler, University of Geneva, Geneva, Switzerland, then imported to the University of Manchester and backcrossed to a C57BL/6J background.

#### Reverbα^Flox2-6^.

We used CRISPR-Cas9 to generate a conditional knockout allele for *Reverbα* (*Nr1d1*), which spans eight exons on mouse chromosome 11. Full details are provided in *SI Appendix*, *Supplementary Text*. We integrated loxP sites at intron 2 and intron 6 (*SI Appendix*, Fig. S3*A*), avoiding any previously described transcriptional regulatory sites (51). For the CRISPR-Cas9, sgRNA were designed using the Sanger Wellcome Trust Sanger Institute (WTSI) website (52) (https://wge.stemcell.sanger.ac.uk//).

#### Reverbα^Flox2-6^Alb^CreERT2^.

In vitro fertilization using Alb^CreERT2^ sperm (C57, a gift from Daniel Metzger and Pierre Chambon, IGBMC, Strasbourg, France) and *Reverbα*^*Flox2-6*^ females was used to derive this line. The original Alb^CreERT2^ generation paper (34) described satisfactory recombination with a tamoxifen regime of 1 mg daily for 5 d (delivered by i.p. injection) and also a lower dose regime of 0.1 mg daily for 5 d (days 1 to 5 of the protocol). We found this lower dose to be effective in inducing *Reverbα* gene and protein knockdown in liver tissue (*SI Appendix*, Fig. S3 *B* and *C*), with effects evident from days 10 to 25 (at least) of the protocol. For tamoxifen treatment, mice were injected with tamoxifen (T5648, Sigma-Aldrich) dissolved in ethanol to a concentration of 10 mg/mL, then in sesame oil (S3547, Sigma-Aldrich) to a final concentration of 1 mg/mL (0.1-mL injection volume). The line was maintained as homozygous for the floxed allele, with Cre^+^ animals used as experimental subjects and Cre^−^ littermates as controls. Both experimental and control animals received tamoxifen treatment to control for tamoxifen effects on gene expression, with mice aged 10 to 11 wk at the start of the protocol. All assessments were carried out on day 15 of the protocol unless otherwise specified.

#### HaloReverbα.

We used CRISPR-Cas9 to generate NR1D1 (REVERBα) N-terminally tagged with a HaloTag fusion protein. Full details are provided in *SI Appendix*, *Supplementary Text*. sgRNA targeting the ATG region of *Nr1d1* were selected using the Sanger WTSI website (52) as before. For our donor repair template, we used the EASI-CRISPR long-ssDNA strategy (53), which comprised the HaloTag gene with linker flanked by 101- and 96-nt homology arms. Validation studies were carried out using homozygotic *HaloReverbα* mice and littermate WT mice.

### In Vivo Phenotyping.

#### Body composition measurement.

Quantitative magnetic resonance imaging was used to assess body composition of live mice (EchoMRI TM Body Composition Analyzer E26-258-MT; “accumulations = 3, water stage = ON”).

#### Blood glucose measurement.

Tail blood was obtained and glucose concentration (mmol/L) read by AVIVA Accu-Chek BM meter and strips (Roche).

#### Food intake measurement.

For 2-hourly food intake recordings, male WT and *Reverbα*^*−/−*^ mice were single-housed in 12 h:12 h light:dark conditions with ad libitum access to normal chow. Cage food was weighed every 2 h over 24 h and intake recorded.

#### Fasting with refeed.

Mice were injected with tamoxifen (as described above) for 5 d. After 14 d, mice were fasted for 16 h (from ZT12 until ZT4), after which time food was replaced. Following 4 h of refeeding, mice were culled and tissues isolated and snap frozen for RNA extraction and analyses.

#### Wheel running.

Mice were individually housed into cages fitted with running wheels and placed into light-tight cabinets (Techniplast). Voluntary wheel-running activity was recorded using ClockLab (Actimetrics). Mice were housed under a 12 h:12 h light:dark (LD) cycle for >7 d, before being switched to constant darkness. After 14 d, mice were exposed to a 1-h light pulse at CT14 and running activity monitored for a further 14 d. In some studies, mice were subsequently exposed to a 1-h light pulse at CT22, with further monitoring after the light pulse.

### Liver Glycogen.

Glycogen levels in liver lysate were quantified by colorimetric EnzyChrom TM Glycogen Assay Kit (E2GN-100, BioAssay Systems), as per manufacturer’s instructions.

### Lipid Extraction and Gas Chromatography.

The Folch chloroform-methanol (2:1; vol/vol) method was used to extract total lipid from tissue lysates (54). To quantify total triacylglyceride, an internal standard (tripentadecanoin glycerol [15:0]) of known concentration was added. Solid-phase extraction was used to separate lipid fractions and fatty acid methyl esters (FAMEs) were prepared as previously described (55). Total triglyceride FAMEs were separated and detected using a 6890N Network GC System (Agilent Technologies) with flame ionization detection, and quantified (micromolar) from the peak area based on their molecular weight. Micromolar quantities were then totalled and each fatty acid was expressed as a percentage of this value (molar percentage; mol%).

### Protein Extraction and Western Blotting.

This was carried out as detailed in *SI Appendix*, *Supplementary Text*. Uncropped blot images can be provided by the corresponding authors.

### RNA Extraction and RT-qPCR.

This was carried out as detailed in *SI Appendix*, *Supplementary Text*.

### RNA-Seq.

We harvested and flash froze liver tissue from adult male mice (*n* = 6 to 8 per group) at ZT8, and extracted total RNA. Individual biological replicates then underwent library preparation and sequencing. Library preparation was performed by the University of Manchester Genomic Technologies Core Facility. A 2200 TapeStation (Agilent Technologies) was used to assess quality and integrity of RNA. The TruSeq Stranded mRNA assay kit (Illumina) was used to prepare libraries, following manufacturer’s instructions. A HiSEq. 4000 instrument (Illumina) was used for paired-end sequencing (76 + 76 cycles, plus indices) of the loaded flow cell. Demultiplexing (allowing for one mismatch) and BCL-to-Fastq conversion was performed using bcl2fastq software (v2.17.1.14) (Illumina).

### RNA-Seq Data Processing and Analysis.

FastQC (v0.11.3) (Babraham Bioinformatics, http://www.bioinformatics.babraham.ac.uk/projects/fastqc/) and FastQ Screen (v 0.9.2) (56) were used to quality check paired-end RNA-seq reads. Adapters and poor quality bases were removed with Trimmomatic (v 0.36) (57). STAR (v2.5.3a) (58) was then used to map reads to the mm10 reference genome. STAR outputs counts per gene (exons) (using the genome annotation GENCODEM16) which were then taken forward to differential expression analysis. For this we used edgeR (59) using published code (60) and employing quasi-likelihood F (QLF) tests. For analysis of published RNA-seq data (37), we downloaded raw counts from GEO, and ran this data through the same edgeR pipeline.

### HaloChIP-Seq.

Liver tissue was collected from homozygous *HaloReverbα* mice at ZT8 (*n* = 3, mixed sex) and flash frozen in liquid nitrogen. For control samples, liver tissue was collected from wild-type mice (related colony, *n* = 2) at ZT8 and from homozygous *HaloReverbα* mice at ZT20 (*n* = 2). A dual cross-linking strategy was employed to capture tethered protein-protein DNA interactions in addition to protein-DNA interactions. Sample preparation is described in detail in *SI Appendix*, *Supplementary Text*. Samples were not pooled; each biological replicate was taken forward individually to library preparation as per manufacturer’s instructions (TruSeq ChIP library preparation kit, Illumina) and paired-end sequencing (Illumina HiSEq. 4000) (mean library size 90m reads per sample).

### ChIP-Seq Data Processing.

We used FastQC (v0.11.7) to perform quality-checking on paired-end ChIP-seq reads. Trimmomatic (v0.38) (57) was used to trim adapters and remove poor quality reads (specifying parameters as: *ILLUMINACLIP:TruSeq3-PE.fa:2:30:10 LEADING:3 TRAILING:3 SLIDINGWINDOW:4:15 MINLEN:36*). Reads were aligned to the reference genome (mm10) with Bowtie2 (v2.3.4.3) (61) (*bowtie2 -p 8 -x mm10 FILENAME.fastq > OUTPUT-FILENAME.sam*). To produce BAM files from the resulting SAM files, we used SAMtools (v1.9) (62) (*view*, *sort*, *index* tools, all with default settings). Finally, to remove duplicate reads, we used Picard (v2.18.14) (https://broadinstitute.github.io/picard/) (*MarkDuplicates* tool, with settings: *REMOVE_DUPLICATES = true ASSUME_SORTED = true*).

### Published ChIP-seq.

The sratoolkit package (v2.9.2) (https://trace.ncbi.nlm.nih.gov/Traces/sra/sra.cgi?view=software) (*fastq-dump* tool) was used to download raw data from the GEO Sequence Read Archive. Published datasets used are specified in *SI Appendix*, Table S9. FASTQ files were processed to BAM files as described above.

### Peak Calling.

Peaks in experimental BAM files were called against control files using MACS2 (v2.1.1.20160309) (63). The following parameters were set:

−f BAMPE−g mm−−keep−dup=1−q 0.01 −−bdg−−SPMR−−verbose 0 .

To find peaks common between datasets, bedtools (v2.27.0) (64) (*intersect* tool) was used (*-u* option set with otherwise default settings).

### Motif Analysis and Peak Annotation.

Motif enrichment analysis was carried out using HOMER (Hypergeometric Optimization of Motif EnRichment) (v4.9) (65), using the findMotifsGenome.pl package (options: *-size 200* and *–mask*). The default settings are to use random genomic regions (within 50 kbp of gene transcription start sites) as the background against which to determine motif enrichment. In addition to this approach, we used the –bg option with background set as peaks called together from ZT6 and ZT10 mouse liver DNase-seq data (28). Motif enrichment, as plotted in Fig. 2*A*, was calculated as the percentage of target regions containing the motif divided by the percentage of background regions containing the motif. To annotate peak locations, the annotatePeaks.pl package was used, using default settings.

### ChIP-Seq Data Visualization.

Integrative Genomics Viewer (v2.4.6) (66) was used to produce visualizations of ChIP-seq data tracks, created with the IGVTools “count” function. deepTools (v2.0) (67) was used to make heatmaps of signal intensity, using the computeMatrix and plotHeatmap functions.

### Integrating RNA-Seq and ChIP-Seq.

As previously described (36), we used a custom Python script to determine enrichment of specified gene clusters at specified distances from REVERBα-binding sites. Peak coordinates are extended in both directions for the given distances (or as defined by TAD/subTAD boundaries). All genes in the mm10 genome whose TSSs fall within these extended coordinates were extracted. The enrichment of a specified gene cluster (derived from RNA-seq analysis) was then calculated using a hypergeometric test. Figs. 3*J* and 4*E* show the *P* values resulting from these tests (−log10 transformed).

### Use of Published HiC Data.

BED files (Browser Extensible Data) of TAD and subTAD coordinates (21) were downloaded from GEO. CrossMap (68) was used to convert the BED files (published in mm9) to mm10. TAD and subTAD coordinates were used alongside peak coordinates and gene clusters in the custom Python package described.

### Detection of Overlapping Published ChIP-Seq Datasets.

HaloREVERBα peaks were uploaded (as a BED file) to the Cistrome DB Toolkit web interface (http://dbtoolkit.cistrome.org/), specifying species as mouse (mm10) and peak number of Cistrome DB sample as 1 k. This tool then employs GIGGLE (29) to compare inputted peak locations with peaks from deposited Cistrome DB datasets (not limited by tissue type) and score search results based on enrichment and significance.

### Pathway Analysis.

Lists of genes with significant differential expression in RNA-seq studies were inputted (as ENTREZ gene identifiers), into the R Bioconductor package Reactome PA (69). We used the enrichPathway tool with settings as follows:

organism=“mouse”,pAdjustMethod=“BH”, maxGSSize=2000, readable=FALSE.

Figs. 3*H* and 4*B* show the 4 pathways with the lowest *P*adj values; *SI Appendix*, Tables S6 and S7 expand this to show the 20 pathways with the lowest *P*adj values.

### Primary Cell Bioluminescence Recording.

Primary lung fibroblasts isolated from homozygous *HaloReverbα* mice and WT littermate controls were transduced with a lentiviral PER2::LUC reporter, and bioluminescence was recorded and analyzed as previously described (70), following synchronization with dexamethasone.

### DR2 Reporter Activity Assay.

HEK293 cells were transfected with the DR2 reporter plasmid (firefly luciferase), either pHT-REVERBα or an empty HaloTag expression construct (pHT), and SV40 renilla luciferase. Luciferase activity was assayed using the Dual-Glo Luciferase Assay System (E2920, Promega) and the Glomax instrument (Promega) as per manufacturer’s instructions. The firefly luciferase signal was normalized using the renilla luciferase signal to control for transfection efficiency.

### Statistics.

Statistical tests used are specified in figure legends, alongside *n* numbers. GraphPad Prism was used to perform these tests, unless otherwise stated. Significance is denoted as *P < 0.05 or **P < 0.01 for tests comparing two or more groups. To define significant differential expression, false discovery rate (FDR) cutoff was set at 0.05. A q-value of 0.01 was set for ChIP-seq peak calling. *N* numbers refer to individual biological replicates (i.e., individual mice). Plots either show individual data points, plus the mean, or show the mean with error bars indicating ± SEM (SEM). The R package ggplot2 and GraphPad Prism were used to produce plots.

## Supplementary Material

Supplementary File

## Data Availability

RNA-seq and ChIP-seq data generated in the course of this study have been uploaded to ArrayExpress. RNA-seq data are available at: https://www.ebi.ac.uk/arrayexpress/experiments/E-MTAB-8402/ (71). ChIP-seq data are available at: https://www.ebi.ac.uk/arrayexpress/experiments/E-MTAB-8413/ (72). Datasets are publicly available.
