# Supplementary material for: Nuclear receptor REVERBα is a state-dependent regulator of liver energy metabolism
Source: Proc Natl Acad Sci U S A. 2020 Sep 28;117(41):25869–79. doi: 10.1073/pnas.2005330117 (PMC7568238; doi:10.1073/pnas.2005330117)
Supplement: Supplementary File [file pnas.2005330117.sapp.pdf]

PNAS
www.pnas.org

Supplementary Information for

*Nuclear receptor REVERBα is a state-dependent regulator of liver energy metabolism*

A. Louise Hunter, Charlotte E. Pelekanou, Antony Adamson, Polly Downton, Nichola J. Barron, Thomas Cornfield, Toryn M. Poolman, Neil Humphreys, Peter S. Cunningham, Leanne Hodson, Andrew S.I. Loudon, Mudassar Iqbal, David A. Bechtold, David W. Ray

Joint corresponding authors: David W. Ray, David A. Bechtold
Email: david.ray@ocdem.ox.ac.uk, david.bechtold@manchester.ac.uk

**This PDF file includes:**

- Supplementary Text
- Figures S1 to S5
- Tables S1 to S9



www.pnas.org/cgi/doi/10.1073/pnas.2005330117

## Supplementary Text

### *Reverbα*$^{Flox2\text{-}6}$ CRISPR generation strategy

No single exon of *Reverbα* (*Nr1d1*) displays suitable criteria for conditional removal (i.e. no unequal splicing phase, small flanking intron sizes for loxP integration). We integrated loxP sites at intron 2 and intron 6 in a two-step process, first achieving a precise integration of the 5' loxP in intron 2, and bred a homozygote colony of this genotype, which were then used as a genetic background to integrate the second 3' loxP. A founder mouse with both 5' and 3' loxP was identified and bred forward for crossing with Cre drivers.

sgRNA were designed using stringent criteria for off-target predictions (guides with mismatch (MM) of 0, 1 or 2 for elsewhere in the genome were discounted, and MM3 were tolerated if predicted off-targets were not exonic). sgRNA were then purchased as crRNA oligos, which were annealed with tracrRNA (both oligos supplied by Sigma-Merck) in sterile, RNase-free injection buffer (TrisHCl 1mM, pH 7.5, EDTA 0.1mM) by combining 2.5 µg crRNA with 5 µg tracrRNA and heating to 95$^{o}$C, which was allowed to slowly cool to room temperature.

For 5' loxP integration, the sgRNA CAGCTAGGGTCTTAGTTACA-*AGG* was used, along with the ssDNA repair template TTTTTTTGTGTGTGTGTGTATGAATAACTGTCAGCTGTCCATCTCCTTCCCAAGTCTACCCataacttcgtatagcatacattatacgaagttat*ggtacc*AGACCCTAGCTGCTGTCAGTACTCCCCAGGAAGCAGCTAGCTCAGGGGTTCCCATGTATGAGATG, where capitals denote the 60bp homology arms, and lowercase bases indicate the loxP site and a KpnI (in italics) for screening purposes. For integration of the 3' loxP in intron 6, the sgRNA AACTCTCCTCTACTCTAGGC-*CGG* was used along with ssDNA2 GGGAAGTCTATCCTCTAGGCTGGTGTTGCCTCCAAACTCAGTGCATCAGAGTAACCAAAGAGACAACTCTCataacttcgtatagcatacattatacgaagttat*ggtacc*CGGAGCTGGTGACCTTCGGTTAAATTACATTCCAGAAGTGGCGGGCACTCCCACCTGCATCC. For embryo microinjection, the annealed sgRNA was complexed with Cas9 protein (New England Biolabs) at room temperature for 10 minutes, before addition of ssDNA donor (final concentrations; sgRNA 20 ng/µl, Cas9 protein 20 ng/µl, ssDNA 50 ng/µl). CRISPR reagents were directly microinjected into B6D2F1 (Envigo) zygote pronuclei using

standard protocols. Zygotes were cultured overnight and the resulting 2-cell embryos surgically implanted into the oviduct of day 0.5 post-coitum pseudopregnant mice. PCR and Sanger sequencing was used to determine correct integration of donor template. The 5' loxP integrated precisely and the full loxP sequence integrated at the 3' site, along with an additional 5bp immediately upstream which has no impact on the mouse model. LoxP sites transmitted to the germline, and a colony was established.

***HaloReverbα* CRISPR generation strategy**

We used CRISPR-Cas9 to generate NR1D1 (REVERBα) N-terminally tagged with a HaloTag fusion protein. As the 3' sequences of *Nr1d1* (*Reverbα*) overlap with the *Thra* gene on the antisense strand, a C-terminal fusion was not possible. 2 sgRNA targeting the ATG region of the *Nr1d1* gene were selected that adhered to our criteria for off-target predictions (as above). The donor repair template, employing the EASI-CRISPR long-ssDNA strategy, comprised the HaloTag gene with linker flanked by 101 and 96nt homology arms. Of note the homology arms contained single nucleotide synonymous nucleotide changes to prevent re-cutting of correctly repaired genomic DNA.

sgRNA sequences (ATGTCTTCACCAGCTGAAAG-CGG and CTCAGTACCTGTGTTGTTAT-TGG) were purchased as crRNA oligos, which were annealed with tracrRNA (Sigma-Merck) in sterile, RNase free injection buffer (TrisHCl 1mM, pH 7.5, EDTA 0.1mM) by combining 2.5 μg crRNA with 5 μg tracrRNA and heating to 95$^{o}$C, which was allowed to slowly cool to room temperature.

For embryo microinjection the annealed sgRNA was complexed with Cas9 protein (New England Biolabs) at room temperature for 10 minutes, before addition of long ssDNA donor (final concentrations; sgRNA 20 ng/μl, Cas9 protein 20 ng/μl, lssDNA 10 ng/μl). CRISPR reagents were directly microinjected into C57BL6/J (Envigo) zygote pronuclei using standard protocols. Zygotes were cultured overnight and the resulting 2 cell embryos surgically implanted into the oviduct of day 0.5 post-coitum pseudopregnant mice. Potential founder mice were screened by PCR, first using primers that flank the sgRNA sites (Cut test F atcctgattgcgaactgcgg, Cut test R gttctcggactgaggggaac), which both identifies editing activity in the form of InDels from NHEJ repair in almost every pup, and can also detect larger products implying HDR. Secondary PCRs used the same homology flanking Cut test primers PCRs in combination with HaloTag primers (HaloTag

R gtaggaggaggtcgggttac, HaloTag F gctgtcgacgctcgagattt). Pups which gave positive products in all three PCR reactions, were sequenced by amplifying again with the cut test F/R primers using high fidelity Phusion polymerase (NEB), gel extracted and subcloned into pCRblunt (Invitrogen) and Sanger sequenced with M13 Forward and Reverse primers. Pups showing perfect sequence integration were bred with C57BL6/J mice to confirm germline transmission. *HaloReverbα* mice were subsequently bred to homozygosity.

**Protein extraction and Western blotting**

Tissue was disrupted in T-PER (Thermo Fisher Scientific), supplemented with Protease Inhibitor Cocktail (Promega), with the FastPrep Lysing Matrix D system (MP Biomedicals). Lysates were benzonase-treated (EMD Millipore) before being spun down (8 minutes at 10,000g, at 4ºC) and the supernatants reserved. Equal masses of protein (75µg for REVERBα detection) were mixed with 4x NuPAGE LDS sample buffer (Invitrogen), NuPAGE sample reducing agent (dithiothreitol) (Invitrogen) and water, then denatured. Proteins were separated on 4-20% Mini-PROTEAN TGX Precast Protein Gels (Bio-Rad), and transferred to nitrocellulose membranes, before membranes were blocked with Protein-Free Blot Blocking Buffer (Azure Biosystems). Membranes were incubated with primary (1:1000 dilution) then secondary antibodies (1:10,000 dilution), with the antibodies being used as follows: rabbit polyclonal anti-REVERBα (NR1D1) (ProteinTech, Cat#14506-1-AP, lot 5745); mouse monoclonal anti-β-ACTIN (ACTB) (ProteinTech, Cat#60008-1, lot 0001084); goat polyclonal anti-Mouse IgG (H+L), CF680R Conjugated (Biotium, Cat#20192-1, lot 11C0414); goat polyclonal anti-Rabbit IgG (H+L), CF770 Conjugated (Biotium. Cat#20078, lot 17C1121); goat polyclonal anti-Rabbit IgG (H+L), DyLight 680 Conjugate, (Cell Signaling Technology, Cat#5366P, lot 7). The LI-COR Odyssey system was used to image blots. Uncropped blot images can be provided by the corresponding authors.

**RNA extraction**

Total RNA was extracted from frozen tissue lysates using TRIzol Reagent (Invitrogen), according to manufacturer's instructions. For RNA-seq, the isopropanol phase of TRIzol extraction was transferred to Reliaprep Tissue Miniprep columns (Promega, USA) and then RNA clean-up and DNase treatment continued as per Reliaprep manufacturer's protocol.

**RT-qPCR**

RNA was DNase treated (RQ1 RNase-Free DNase, Promega, USA) then converted to cDNA using the High Capacity RNA-to-cDNA kit (Applied Biosystems). GoTaq qPCR Master Mix (Promega, USA) and primers listed in SI Appendix, Table S8 were then used to perform qPCR, using the Step One Plus (Applied Biosystems) qPCR system. The ΔΔCT method was used to calculate relative gene expression, normalised using the geometric mean of the housekeeping genes *Hprt, Ppib* and *Actb*, and then calculating fold difference of expression relative to control groups.

**HaloChIP-seq sample preparation**

Frozen liver tissue was first homogenised in 2mM disuccinimidyl glutarate (DSG) (20593, Thermo Fisher Scientific) using a handheld TissueRuptor (Qiagen) and rotated at room temperature for 15 minutes. After being spun down, the cell pellet was resuspended in 1% formaldehyde-PBS, rotated for a further 15 minutes, before glycine quenching with Stop Solution (Active Motif ChIP-IT High Sensitivity kit 53040). The cell pellet was washed and dounce-homogenised as per the standard Active Motif ChIP-IT kit instructions. It was then resuspended in 650μl of Mammalian Cell Lysis Buffer (Promega HaloChIP kit G9410) supplemented with Protease Inhibitor Cocktail (G6521, Promega) and incubated on ice for 15 minutes before sonication at 20% amplitude for 8 cycles (30s on, 30s off, with 2 minutes of 'on' time per cycle), using the Active Motif EpiShear Probe Sonicator. Chromatin concentration was quantified with the Qubit BR dsDNA assay (Q32850, Thermo Fisher Scientific) and shearing efficacy checked with TapeStation electrophoresis (Agilent Technologies). For the pull-down step, 100μg chromatin, in a volume of 650μl Mammalian Cell Lysis Buffer, was rotated at room temperature with prepared HaloLink Resin (Promega) as per the Promega HaloChIP kit protocol. Wash and elution steps were also carried out as per the same manufacturer's protocol, incorporating the recommended LiCl buffer wash step. ChIP DNA was cleaned up with the MinElute PCR Purification Kit (28004, Qiagen) and quantified with the Qubit HS dsDNA assay (Q32854, Thermo Fisher Scientific).

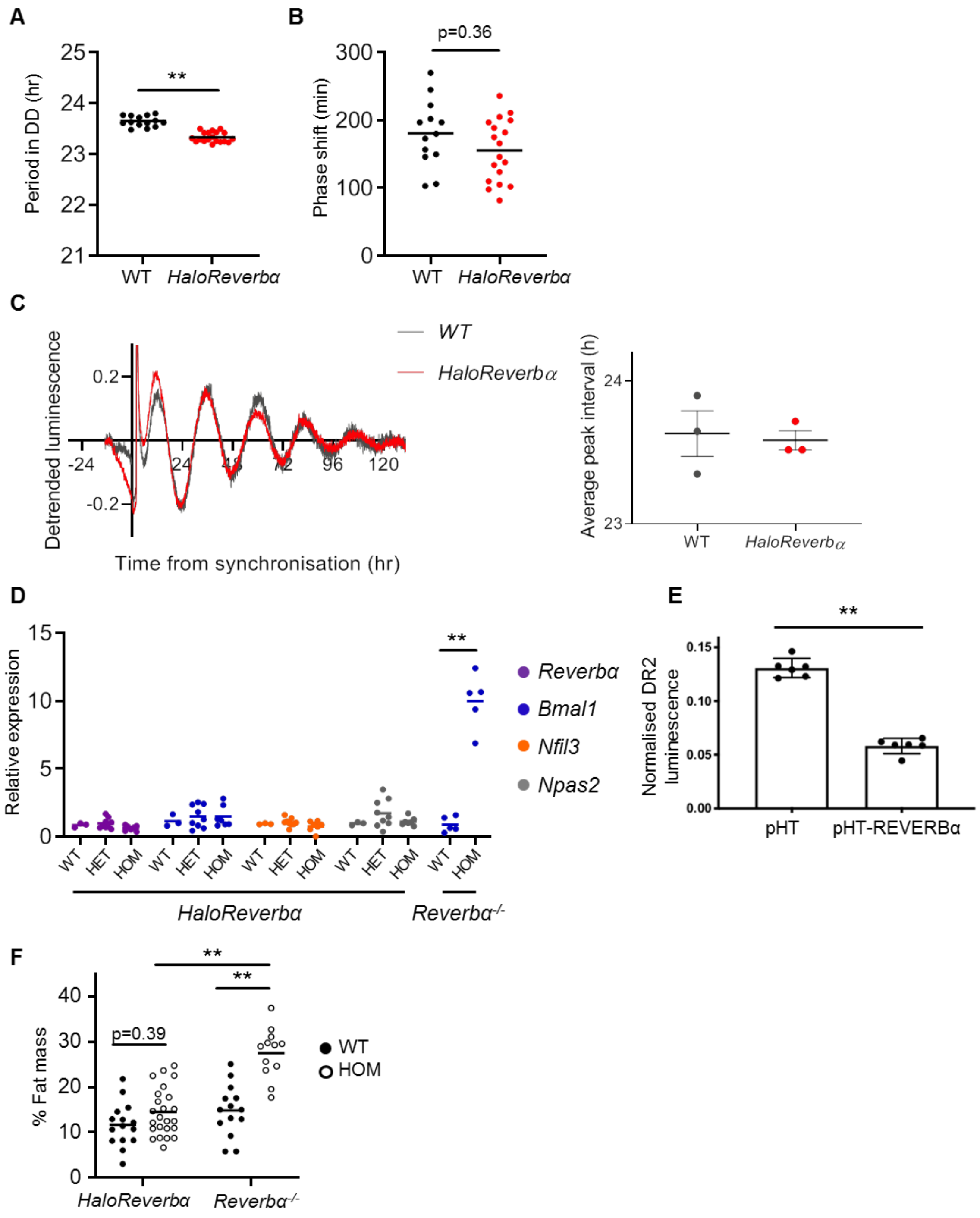


**Fig. S1. A.** Length of free-running period for WT and *HaloReverbα* mice in constant darkness (DD). **B.** Shift response to a 1hr light pulse delivered at CT14 in WT and *HaloReverbα* mice. n=13-17 per group. **C.** Representative bioluminescence recordings (left) collected from primary lung fibroblasts transduced with a PER2::LUC lentiviral reporter. Time zero indicates synchronisation with dexamethasone treatment. Right-hand plot shows circadian period of PER2::LUC bioluminescence, in WT and *HaloReverbα* primary cells. Each point represents

individual animals, each multiple (>3) replicates. **D.** Relative expression (as measured by qPCR) of clock genes *Reverbα*, *Bmal1*, *Nfil3*, *Npas2* in liver tissue from WT, heterozygous and homozygous *HaloReverbα* mice at ZT8. Liver *Bmal1* expression from WT and *Reverbα*$^{-/-}$ mice shown for comparison. Relative expression calculated separately for each gene. n=3-9 per group. **E.** HaloREVERBα-mediated repression of DR2 element-driven reporter activity. Expression of HaloREVERBα (pHT-REVERBα) in HEK293 cells causes a significant repression of DR2-site reporter activity, relative to expression of HaloTag (pHT) alone. n=6 per group (biological replicates). **F.** Percentage fat mass (of total body mass) of 13-week old *HaloReverbα* and *Reverbα*$^{-/-}$ male mice (empty circles) and their respective WT littermate controls (filled circles). n=12-24 per group.

Data displayed as individual data points plus mean (A,B,D,F) +/- SEM (C,E); unpaired t-tests (A-E), two-way ANOVA with Tukey's multiple comparisons tests (F). **P<0.01.

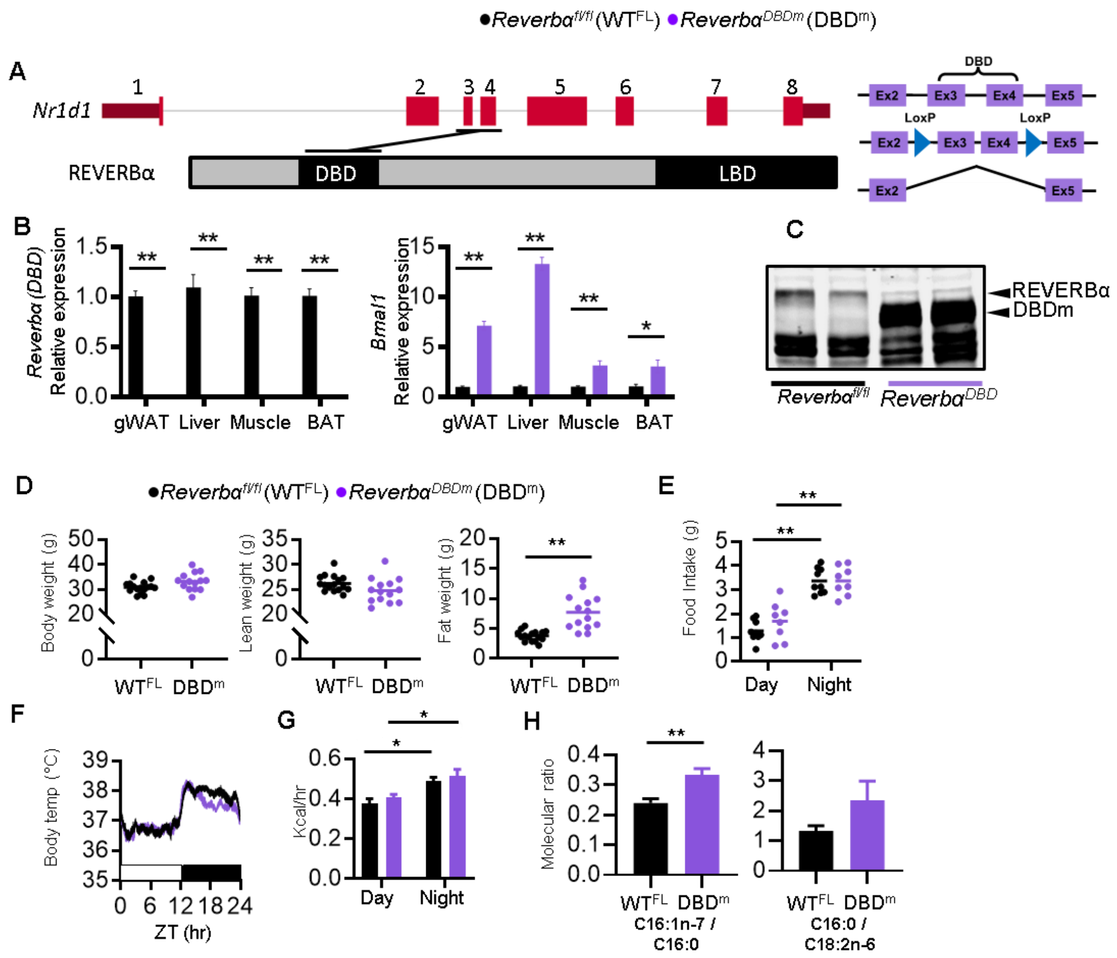


**Fig. S2. A.** Schematic of the *Nr1d1/Reverbα* gene and protein, showing targeting of DNA-binding domain (*Reverbα*$^{DBDm}$). **B.** qPCR validation of *Reverbα*$^{DBDm}$ targeting leading to a loss of *Reverbα* expression and up-regulation of *Bmal1* (qPCR primers target the DNA binding domain region of *Reverbα*). (n=6 per group) **C.** As previously reported (20), *Reverbα*$^{DBDm}$ produces an over-expressed hypomorph of REVERBα shown here by Western blot analysis of liver tissue. **D.** Despite similar body weights, *Reverbα*$^{DBD}$ mice exhibit a significant increase in fat mass relative to littermate controls (13 week-old males, n = 14-16 per group). **E-G.** This adiposity phenotype was not accompanied by altered daily food intake (**E**), body temperature (**F**), or energy expenditure (**G**). All mice showed robust diurnal rhythms in physiological parameters. n=8-9 per group). **H.** Analyses of fatty acid (FA) composition in livers of *Reverbα*$^{DBD}$ mice and littermate controls. FA desaturation is reflected by C16:1n-7/C16:0 ratio, *de novo* lipogenesis by C16:0/C18:2n ratio. n=5-6 per group.

Data displayed as individual data points with line at mean (D, E) or mean +/- SEM (B, G, H); two-way ANOVA with Tukey's multiple comparisons tests (E, G) or unpaired t-tests (B, D, H) with correction for multiple testing (B). *P<0.05, **P<0.01.

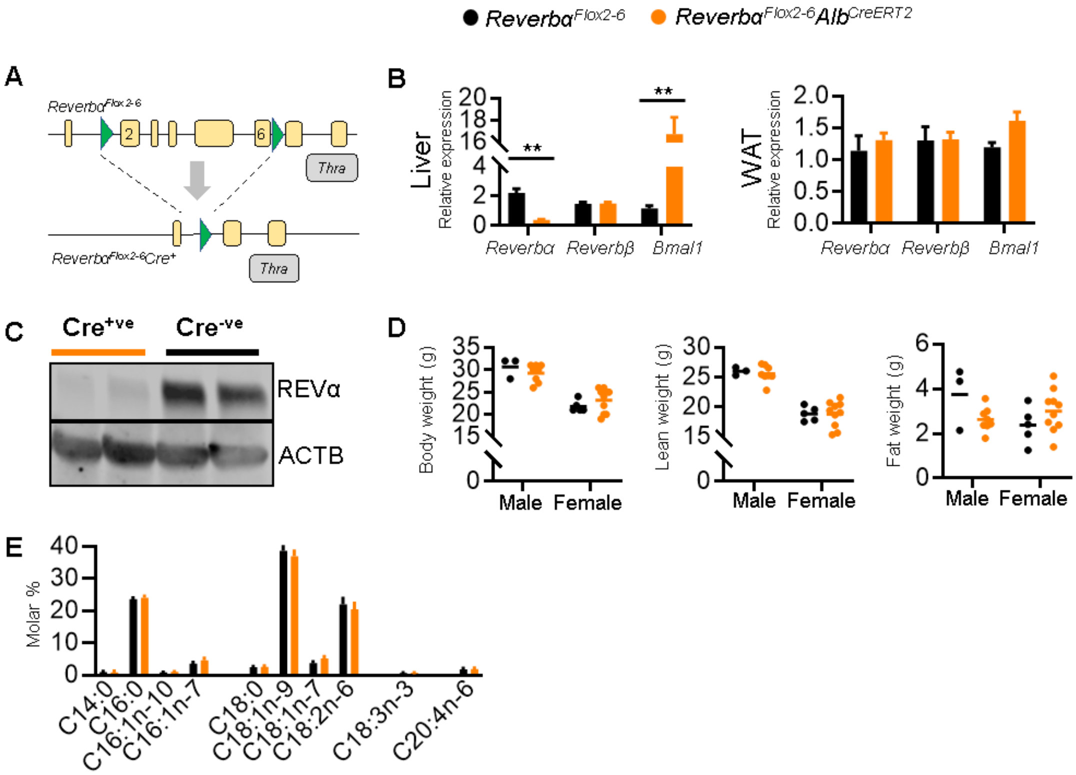


**Fig. S3. A.** Targeting strategy for LoxP site integration flanking exons 2 to 6 of the *Nr1d1* (*Reverbα*) locus. **B-C.** Liver-specific deletion of REVERBα achieved with tamoxifen-treatment of *Reverbα*$^{Flox2\text{-}6}$*Alb*$^{CreERT2}$ mice, as confirmed by *Reverbα, Reverbβ* and *Bmal1* gene expression in liver and WAT (**B**; n=8-16 per group), and Western blot analysis of liver REVERBα (**C**). **D.** Body weight and body composition of male and female *Reverbα*$^{Flox2\text{-}6}$*Alb*$^{CreERT2}$ mice and *Reverbα*$^{Flox2\text{-}6}$ littermate controls (12-14 week-old animals, n = 3-10 per group, profiled on day 25 after the start of tamoxifen treatment). **E.** Fatty acid composition analysis (molar percentages) of *Reverbα*$^{Flox2\text{-}6}$*Alb*$^{CreERT2}$ liver and *Reverbα*$^{Flox2\text{-}6}$ littermate controls (n=6-8 per group, 12-14 week-old males and females, 15 days after the start of tamoxifen treatment).

Data shown as mean +/-SEM (B,E), or as individual data points with line at mean (D), **P<0.01, unpaired t-tests with correction for multiple testing (B,E); two-way ANOVA with Tukey's multiple comparisons tests (D).

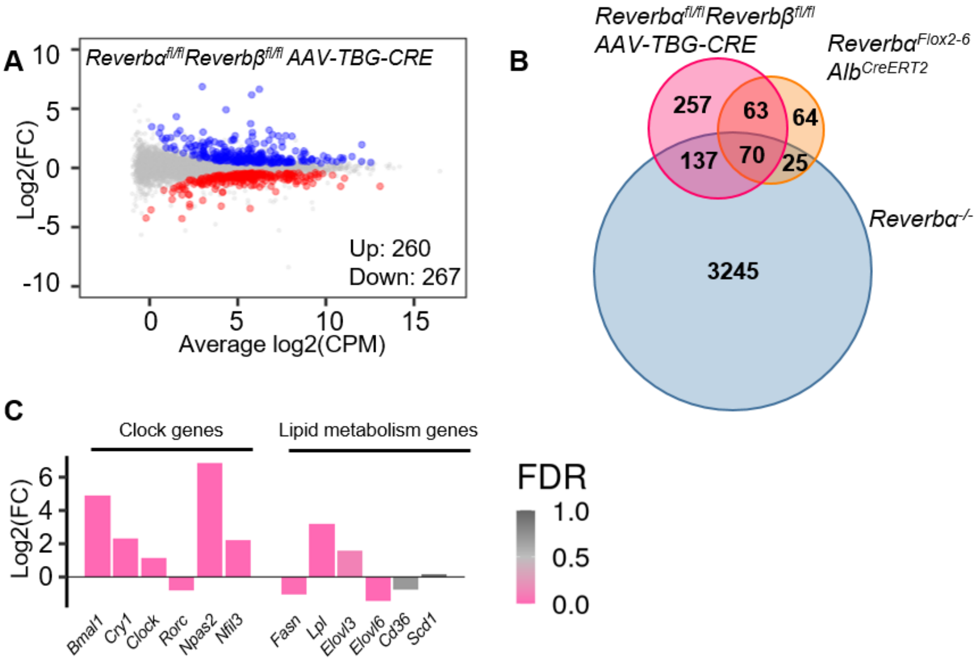


**Fig. S4. A.** Mean difference (MD) plot of ZT10 liver RNA-seq from *Reverbα*$^{fl/fl}$*Reverbβ*$^{fl/fl}$ *AAV-TBG-CRE* mice (relative to *Reverbα*$^{fl/fl}$*Reverbβ*$^{fl/fl}$ *AAV-TBG-GFP* controls); genes with significant differential expression (FDR<0.05) highlighted in colour (up-regulated in blue, down-regulated in red). Raw data from Guan et al. 2020 (37). **B.** Venn diagram showing overlap of significantly differentially expressed genes in liver RNA-seq from the three models: *Reverbα*$^{fl/fl}$*Reverbβ*$^{fl/fl}$ *AAV-TBG-CRE*, *Reverbα*$^{Flox2-6}$*Alb*$^{CreERT2}$, *Reverbα*$^{-/-}$. **C.** Fold-change (FC) and false discovery rate (FDR) of selected circadian clock and lipid metabolism genes from RNA-seq experiment in **A**.

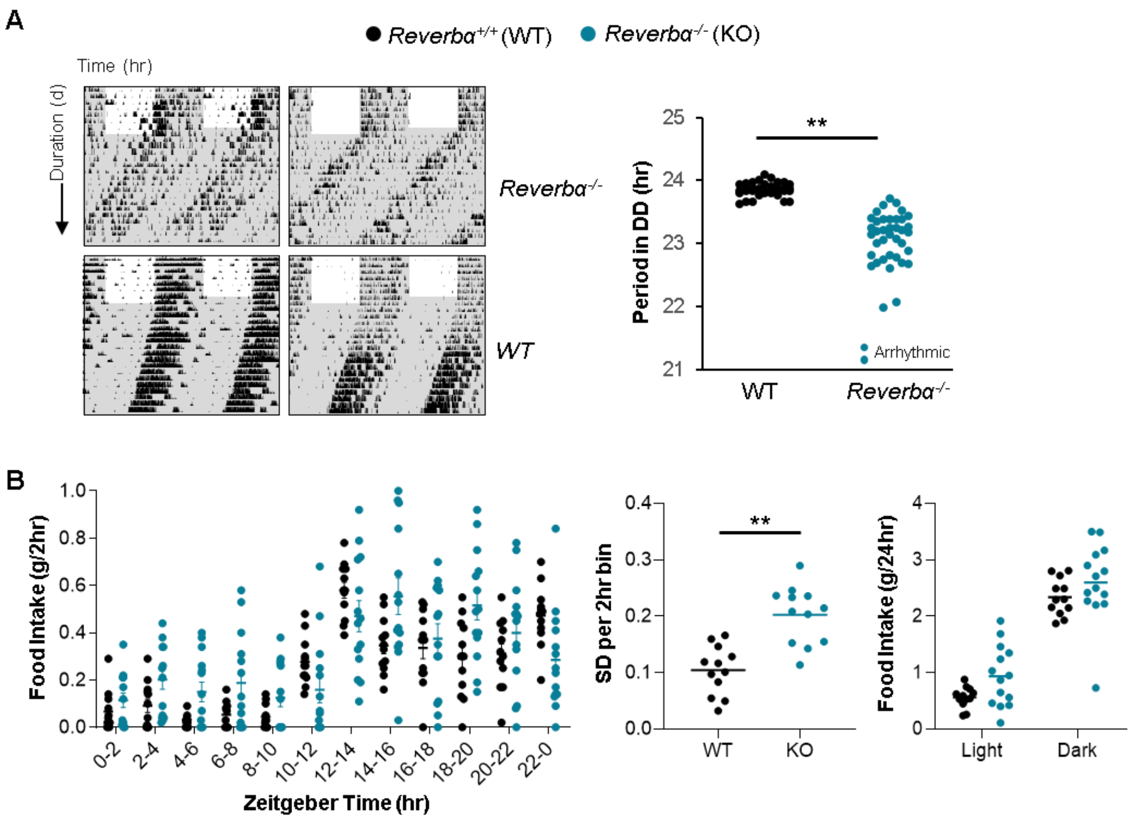


**Fig. S5. A.** Circadian wheel-running activity of *Reverbα*$^{-/-}$ mice and WT littermate controls under 12hr:12hr light:dark (LD) and constant darkness (DD) conditions. Representative actigrams shown on left, length of free-running period quantified on right. n=36-39 per group. **B.** Circadian patterns of food intake in single-housed *Reverbα*$^{-/-}$ (teal) and WT mice (black). Left plot shows food intake per two hour time window, middle plot shows standard deviation of data within each window, right plot shows total food intake over 24 hours. n=12-14 per group.

Data shown as individual data points plus mean +/- SEM, or with mean alone. **P<0.01, unpaired t-test (A, B).

**Table S1.** Known motif analysis of HaloREVERBα sites, random background

# Homer Known Motif Enrichment Results (HOMER_high-confidence_peaks/)

Homer *de novo* Motif Results
Gene Ontology Enrichment Results
Known Motif Enrichment Results (txt file)
Total Target Sequences = 7772, Total Background Sequences = 41747

| Rank | Motif | Name | P-value | log P-pvalue | q-value (Benjamini) | # Target Sequences with Motif | % of Targets Sequences with Motif | # Background Sequences with Motif | % of Background Sequences with Motif | Motif File | SVG |
|---|---|---|---|---|---|---|---|---|---|---|---|
| 1 | 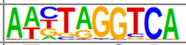 | RORgt(NR)/EL4-RORgt.Flag-ChIP-Seq(GSE56019)/Homer | 1e-282 | -6.500e+02 | 0.0000 | 551.0 | 7.09% | 396.6 | 0.95% | motif file (matrix) | svg |
| 2 | 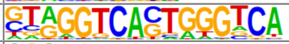 | Reverb(NR),DR2/RAW-Reverba.biotin-ChIP-Seq(GSE45914)/Homer | 1e-182 | -4.207e+02 | 0.0000 | 482.0 | 6.20% | 496.8 | 1.19% | motif file (matrix) | svg |
| 3 | 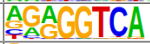 | COUP-TFII(NR)/Artia-Nr2f2-ChIP-Seq(GSE46497)/Homer | 1e-153 | -3.525e+02 | 0.0000 | 2138.0 | 27.49% | 6556.8 | 15.70% | motif file (matrix) | svg |
| 4 | 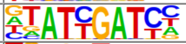 | HNF6(Homeobox)/Liver-Hnf6-ChIP-Seq(ERP000394) /Homer | 1e-149 | -3.442e+02 | 0.0000 | 812.0 | 10.44% | 1536.3 | 3.68% | motif file (matrix) | svg |
| 5 | 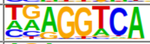 | THRb(NR)/Liver-NR1A2-ChIP-Seq(GSE52613)/Homer | 1e-145 | -3.339e+02 | 0.0000 | 4040.0 | 51.95% | 15703.4 | 37.59% | motif file (matrix) | svg |
| 6 | 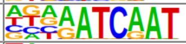 | Cux2(Homeobox)/Liver-Cux2-ChIP-Seq(GSE35985)/Homer | 1e-133 | -3.082e+02 | 0.0000 | 681.0 | 8.76% | 1229.3 | 2.94% | motif file (matrix) | svg |
| 7 | 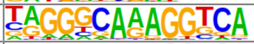 | RXR(NR),DR1/3T3L1-RXR-ChIP-Seq(GSE13511)/Homer | 1e-111 | -2.566e+02 | 0.0000 | 1446.0 | 18.60% | 4227.2 | 10.12% | motif file (matrix) | svg |
| 8 | 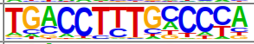 | PPARE(NR),DR1/3T3L1-Pparg-ChIP-Seq(GSE13511) /Homer | 1e-106 | -2.456e+02 | 0.0000 | 1253.0 | 16.11% | 3512.2 | 8.41% | motif file (matrix) | svg |
| 9 | 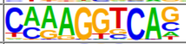 | Erra(NR)/HepG2-Erra-ChIP-Seq(GSE31477)/Homer | 1e-101 | -2.338e+02 | 0.0000 | 2376.0 | 30.56% | 8460.0 | 20.25% | motif file (matrix) | svg |
| 10 | 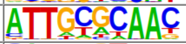 | CEBP(bZIP)/ThioMac-CEBPb-ChIP-Seq(GSE21512)/Homer | 1e-89 | -2.063e+02 | 0.0000 | 803.0 | 10.33% | 1985.1 | 4.75% | motif file (matrix) | svg |
| 11 | 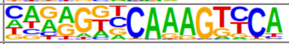 | HNF4a(NR),DR1/HepG2-HNF4a-ChIP-Seq(GSE25021) /Homer | 1e-81 | -1.877e+02 | 0.0000 | 717.0 | 9.22% | 1753.5 | 4.20% | motif file (matrix) | svg |
| 12 | 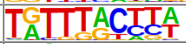 | FOXM1(Forkhead)/MCF7-FOXM1-ChIP-Seq(GSE72977) /Homer | 1e-78 | -1.813e+02 | 0.0000 | 1089.0 | 14.00% | 3220.9 | 7.71% | motif file (matrix) | svg |
| 13 | 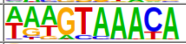 | FOXA1(Forkhead)/LNCAP-FOXA1-ChIP-Seq(GSE27824) /Homer | 1e-76 | -1.760e+02 | 0.0000 | 1215.0 | 15.62% | 3772.8 | 9.03% | motif file (matrix) | svg |
| 14 | 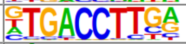 | Esrrb(NR)/mES-Esrrb-ChIP-Seq(GSE11431)/Homer | 1e-71 | -1.646e+02 | 0.0000 | 862.0 | 11.09% | 2412.1 | 5.77% | motif file (matrix) | svg |
| 15 | 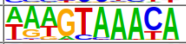 | FOXA1(Forkhead)/MCF7-FOXA1-ChIP-Seq(GSE26831) /Homer | 1e-67 | -1.562e+02 | 0.0000 | 995.0 | 12.80% | 2993.6 | 7.17% | motif file (matrix) | svg |
| 16 | 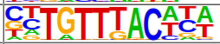 | Foxa2(Forkhead)/Liver-Foxa2-ChIP-Seq(GSE25694) /Homer | 1e-52 | -1.215e+02 | 0.0000 | 842.0 | 10.83% | 2594.8 | 6.21% | motif file (matrix) | svg |
| 17 | 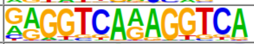 | TR4(NR),DR1/Hela-TR4-ChIP-Seq(GSE24685)/Homer | 1e-52 | -1.204e+02 | 0.0000 | 261.0 | 3.36% | 461.3 | 1.10% | motif file (matrix) | svg |
| 18 | 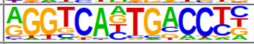 | FXR(NR),IR1/Liver-FXR-ChIP-Seq(Chong_et_al.)/Homer | 1e-50 | -1.169e+02 | 0.0000 | 550.0 | 7.07% | 1466.8 | 3.51% | motif file (matrix) | svg |
| 19 | 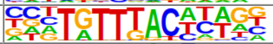 | Foxa3(Forkhead)/Liver-Foxa3-ChIP-Seq(GSE77670) /Homer | 1e-50 | -1.159e+02 | 0.0000 | 396.0 | 5.09% | 912.9 | 2.19% | motif file (matrix) | svg |
| 20 | 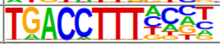 | Nur77(NR)/K562-NR4A1-ChIP-Seq(GSE31363)/Homer | 1e-48 | -1.128e+02 | 0.0000 | 300.0 | 3.86% | 603.8 | 1.45% | motif file (matrix) | svg |
| 21 | 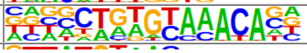 | Fox:Ebox(Forkhead,bHLH)/Panc1-Foxa2-ChIP-Seq(GSE47459)/Homer | 1e-45 | -1.039e+02 | 0.0000 | 985.0 | 12.67% | 3330.9 | 7.97% | motif file (matrix) | svg |

**Table S2.** Known motif analysis of HaloREVERBα sites, DNase background

# Homer Known Motif Enrichment Results (ZT6_ZT10_DNase)

Homer *de novo* Motif Results
Gene Ontology Enrichment Results
Known Motif Enrichment Results (txt file)
Total Target Sequences = 7772, Total Background Sequences = 79824

| Rank | Motif | Name | P-value | log P-pvalue | q-value (Benjamini) | # Target Sequences with Motif | % of Targets Sequences with Motif | # Background Sequences with Motif | % of Background Sequences with Motif | Motif File | SVG |
|---|---|---|---|---|---|---|---|---|---|---|---|
| 1 | 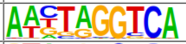 | RORgt(NR)/EL4-RORgt.Flag-ChIP-Seq(GSE56019)/Homer | 1e-226 | -5.214e+02 | 0.0000 | 551.0 | 7.09% | 997.8 | 1.25% | motif file (matrix) | svg |
| 2 | 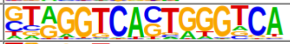 | Reverb(NR),DR2/RAW-Reverba.biotin-ChIP-Seq(GSE45914)/Homer | 1e-179 | -4.127e+02 | 0.0000 | 482.0 | 6.20% | 970.9 | 1.21% | motif file (matrix) | svg |
| 3 | 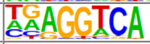 | THRb(NR)/Liver-NR1A2-ChIP-Seq(GSE52613)/Homer | 1e-40 | -9.288e+01 | 0.0000 | 4040.0 | 51.95% | 35535.3 | 44.39% | motif file (matrix) | svg |
| 4 | 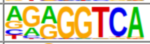 | COUP-TFII(NR)/Artia-Nr2f2-ChIP-Seq(GSE46497)/Homer | 1e-29 | -6.901e+01 | 0.0000 | 2138.0 | 27.49% | 17582.1 | 21.96% | motif file (matrix) | svg |
| 5 | 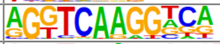 | RAR:RXR(NR),DR5/ES-RAR-ChIP-Seq(GSE56893)/Homer | 1e-21 | -4.890e+01 | 0.0000 | 238.0 | 3.06% | 1235.7 | 1.54% | motif file (matrix) | svg |
| 6 | 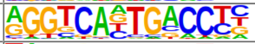 | FXR(NR),IR1/Liver-FXR-ChIP-Seq(Chong_et_al.)/Homer | 1e-20 | -4.609e+01 | 0.0000 | 550.0 | 7.07% | 3753.3 | 4.69% | motif file (matrix) | svg |
| 7 | 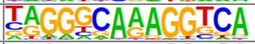 | RXR(NR),DR1/3T3L1-RXR-ChIP-Seq(GSE13511)/Homer | 1e-15 | -3.538e+01 | 0.0000 | 1446.0 | 18.60% | 12185.0 | 15.22% | motif file (matrix) | svg |
| 8 | 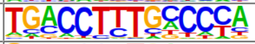 | PPARE(NR),DR1/3T3L1-Pparg-ChIP-Seq(GSE13511)/Homer | 1e-14 | -3.299e+01 | 0.0000 | 1253.0 | 16.11% | 10453.1 | 13.06% | motif file (matrix) | svg |
| 9 | 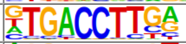 | Esrrb(NR)/mES-Esrrb-ChIP-Seq(GSE11431)/Homer | 1e-9 | -2.260e+01 | 0.0000 | 862.0 | 11.09% | 7183.5 | 8.97% | motif file (matrix) | svg |
| 10 | 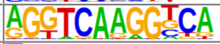 | RARg(NR)/ES-RARg-ChIP-Seq(GSE30538)/Homer | 1e-8 | -2.029e+01 | 0.0000 | 80.0 | 1.03% | 390.9 | 0.49% | motif file (matrix) | svg |
| 11 | 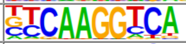 | Nr5a2(NR)/Pancreas-LRH1-ChIP-Seq(GSE34295)/Homer | 1e-8 | -1.903e+01 | 0.0000 | 773.0 | 9.94% | 6492.2 | 8.11% | motif file (matrix) | svg |
| 12 | 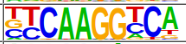 | Nr5a2(NR)/mES-Nr5a2-ChIP-Seq(GSE19019)/Homer | 1e-7 | -1.705e+01 | 0.0000 | 584.0 | 7.51% | 4808.3 | 6.01% | motif file (matrix) | svg |
| 13 | 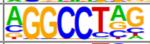 | ZNF711(Zf)/SHSY5Y-ZNF711-ChIP-Seq(GSE20673)/Homer | 1e-6 | -1.565e+01 | 0.0000 | 1654.0 | 21.27% | 15178.9 | 18.96% | motif file (matrix) | svg |
| 14 | 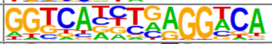 | THRa(NR)/C17.2-THRa-ChIP-Seq(GSE38347)/Homer | 1e-6 | -1.447e+01 | 0.0000 | 464.0 | 5.97% | 3795.9 | 4.74% | motif file (matrix) | svg |
| 15 | 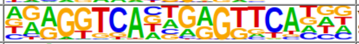 | VDR(NR),DR3/GM10855-VDR+vitD-ChIP-Seq(GSE22484)/Homer | 1e-6 | -1.400e+01 | 0.0000 | 229.0 | 2.94% | 1692.6 | 2.11% | motif file (matrix) | svg |
| 16 | 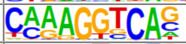 | Erra(NR)/HepG2-Erra-ChIP-Seq(GSE31477)/Homer | 1e-6 | -1.383e+01 | 0.0000 | 2376.0 | 30.56% | 22500.1 | 28.11% | motif file (matrix) | svg |
| 17 | 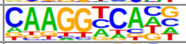 | SF1(NR)/H295R-Nr5a1-ChIP-Seq(GSE44220)/Homer | 1e-4 | -9.549e+00 | 0.0015 | 489.0 | 6.29% | 4236.9 | 5.29% | motif file (matrix) | svg |
| 18 | 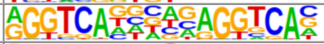 | RAR:RXR(NR),DR5/ES-RAR-ChIP-Seq(GSE56893)/Homer | 1e-3 | -8.712e+00 | 0.0033 | 52.0 | 0.67% | 309.7 | 0.39% | motif file (matrix) | svg |
| 19 | 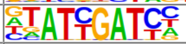 | HNF6(Homeobox)/Liver-Hnf6-ChIP-Seq(ERP000394)/Homer | 1e-3 | -8.006e+00 | 0.0064 | 812.0 | 10.44% | 7443.7 | 9.30% | motif file (matrix) | svg |
| 20 | 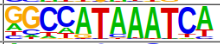 | Hoxc9(Homeobox)/Ainv15-Hoxc9-ChIP-Seq(GSE21812)/Homer | 1e-3 | -7.888e+00 | 0.0068 | 343.0 | 4.41% | 2935.7 | 3.67% | motif file (matrix) | svg |
| 21 | 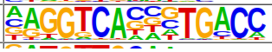 | ERE(NR),IR3/MCF7-ERa-ChIP-Seq(Unpublished)/Homer | 1e-3 | -7.864e+00 | 0.0068 | 262.0 | 3.37% | 2179.4 | 2.72% | motif file (matrix) | svg |

**Table S3.** *De novo* motif analysis of HaloREVERBα sites, random background

# Homer *de novo* Motif Results (HOMER_high-confidence_peaks//)

Known Motif Enrichment Results
Gene Ontology Enrichment Results
If Homer is having trouble matching a motif to a known motif, try copy/pasting the matrix file into STAMP
More information on motif finding results: HOMER | Description of Results | Tips
Total target sequences = 7775
Total background sequences = 41747
* - possible false positive

| Rank | Motif | P-value | log P-pvalue | % of Targets | % of Background | STD(Bg STD) | Best Match/Details | Motif File |
|---|---|---|---|---|---|---|---|---|
| 1 | 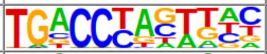 | 1e-470 | -1.083e+03 | 17.25% | 3.74% | 53.0bp (57.2bp) | RORgt(NR)/EL4-RORgt.Flag-ChIP-Seq(GSE56019)/Homer(0.939) More Information \| Similar Motifs Found | motif file (matrix) |
| 2 | 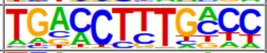 | 1e-147 | -3.403e+02 | 20.91% | 10.78% | 55.3bp (56.7bp) | RXR(NR),DR1/3T3L1-RXR-ChIP-Seq(GSE13511)/Homer(0.941) More Information \| Similar Motifs Found | motif file (matrix) |
| 3 | 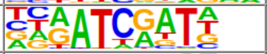 | 1e-137 | -3.170e+02 | 7.81% | 2.37% | 55.0bp (59.0bp) | HNF6(Homeobox)/Liver-Hnf6-ChIP-Seq(ERP000394)/Homer(0.936) More Information \| Similar Motifs Found | motif file (matrix) |
| 4 | 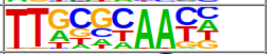 | 1e-108 | -2.508e+02 | 12.38% | 5.69% | 54.9bp (60.9bp) | CEBPE/MA0837.1/Jaspar(0.940) More Information \| Similar Motifs Found | motif file (matrix) |
| 5 | 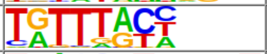 | 1e-68 | -1.570e+02 | 26.58% | 18.49% | 54.9bp (60.5bp) | FOXD2/MA0847.1/Jaspar(0.963) More Information \| Similar Motifs Found | motif file (matrix) |
| 6 | 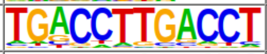 | 1e-64 | -1.488e+02 | 4.84% | 1.76% | 56.2bp (59.2bp) | RARg(NR)/ES-RARg-ChIP-Seq(GSE30538)/Homer(0.968) More Information \| Similar Motifs Found | motif file (matrix) |
| 7 | 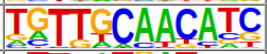 | 1e-32 | -7.596e+01 | 1.74% | 0.50% | 55.1bp (55.8bp) | CEBP:AP1(bZIP)/ThioMac-CEBPb-ChIP-Seq(GSE21512)/Homer(0.894) More Information \| Similar Motifs Found | motif file (matrix) |
| 8 | 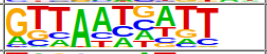 | 1e-30 | -6.976e+01 | 3.12% | 1.36% | 53.6bp (59.3bp) | HNF1b(Homeobox)/PDAC-HNF1B-ChIP-Seq(GSE64557)/Homer(0.871) More Information \| Similar Motifs Found | motif file (matrix) |
| 9 | 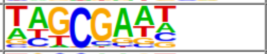 | 1e-28 | -6.675e+01 | 31.03% | 25.35% | 56.4bp (61.0bp) | PB0034.1_Irf4_1/Jaspar(0.637) More Information \| Similar Motifs Found | motif file (matrix) |
| 10 | 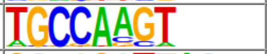 | 1e-23 | -5.463e+01 | 6.85% | 4.32% | 55.0bp (59.5bp) | NFIA/MA0670.1/Jaspar(0.893) More Information \| Similar Motifs Found | motif file (matrix) |
| 11 | 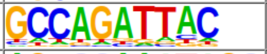 | 1e-20 | -4.669e+01 | 6.57% | 4.27% | 55.3bp (57.0bp) | Hand1::Tcf3/MA0092.1/Jaspar(0.701) More Information \| Similar Motifs Found | motif file (matrix) |
| 12 | 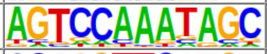 | 1e-19 | -4.486e+01 | 1.25% | 0.42% | 51.8bp (56.7bp) | PB0134.1_Hnf4a_2/Jaspar(0.648) More Information \| Similar Motifs Found | motif file (matrix) |
| 13 | 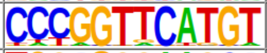 | 1e-17 | -4.053e+01 | 0.21% | 0.01% | 58.9bp (20.8bp) | PB0153.1_Nr2f2_2/Jaspar(0.580) More Information \| Similar Motifs Found | motif file (matrix) |
| 14 | 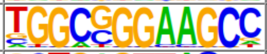 | 1e-16 | -3.826e+01 | 0.32% | 0.03% | 53.3bp (55.3bp) | E2F1(E2F)/Hela-E2F1-ChIP-Seq(GSE22478)/Homer(0.808) More Information \| Similar Motifs Found | motif file (matrix) |
| 15 | 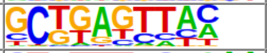 | 1e-15 | -3.673e+01 | 1.40% | 0.57% | 59.5bp (55.3bp) | Nrf2(bZIP)/Lymphoblast-Nrf2-ChIP-Seq(GSE37589)/Homer(0.733) More Information \| Similar Motifs Found | motif file (matrix) |
| 16 | 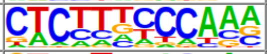 | 1e-15 | -3.467e+01 | 0.48% | 0.09% | 56.0bp (65.1bp) | Rbpj1(?)/Panc1-Rbpj1-ChIP-Seq(GSE47459)/Homer(0.640) More Information \| Similar Motifs Found | motif file (matrix) |
| 17 | 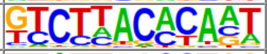 | 1e-14 | -3.388e+01 | 0.18% | 0.01% | 57.5bp (30.6bp) | PB0168.1_Sox14_2/Jaspar(0.687) More Information \| Similar Motifs Found | motif file (matrix) |
| 18 | 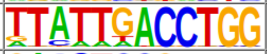 | 1e-13 | -3.065e+01 | 0.17% | 0.01% | 59.8bp (14.2bp) | ZEB1(Zf)/PDAC-ZEB1-ChIP-Seq(GSE64557)/Homer(0.699) More Information \| Similar Motifs Found | motif file (matrix) |
| 19 | 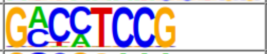 | 1e-12 | -2.860e+01 | 4.67% | 3.15% | 55.3bp (63.2bp) | ETV5/MA0765.1/Jaspar(0.751) More Information \| Similar Motifs Found | motif file (matrix) |
| 20 * | 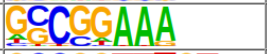 | 1e-11 | -2.755e+01 | 4.54% | 3.07% | 54.9bp (60.9bp) | Elk4(ETS)/Hela-Elk4-ChIP-Seq(GSE31477)/Homer(0.848) More Information \| Similar Motifs Found | motif file (matrix) |
| [illegible] | [illegible] | [illegible] | [illegible] | [illegible] | [illegible] | [illegible] | TFCP2/MA0145.3/Jaspar(0.692) | [illegible] |

**Table S4.** *De novo* motif analysis of HaloREVERBα sites, DNase background

# Homer *de novo* Motif Results (ZT6_ZT10_DNase_size200_denovo/)

Known Motif Enrichment Results
Gene Ontology Enrichment Results
If Homer is having trouble matching a motif to a known motif, try copy/pasting the matrix file into STAMP
More information on motif finding results: HOMER | Description of Results | Tips
Total target sequences = 7775
Total background sequences = 80034
* - possible false positive

| Rank | Motif | P-value | log P-pvalue | % of Targets | % of Background | STD(Bg STD) | Best Match/Details | Motif File |
|---|---|---|---|---|---|---|---|---|
| 1 | 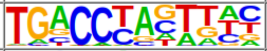 | 1e-371 | -8.548e+02 | 17.54% | 4.78% | 52.8bp (57.2bp) | RORgt(NR)/EL4-RORgt.Flag-ChIP-Seq(GSE56019)/Homer(0.942) More Information \| Similar Motifs Found | motif file (matrix) |
| 2 | 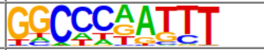 | 1e-32 | -7.563e+01 | 9.21% | 5.77% | 56.3bp (58.4bp) | ZNF711(Zf)/SHSY5Y-ZNF711-ChIP-Seq(GSE20673)/Homer(0.577) More Information \| Similar Motifs Found | motif file (matrix) |
| 3 | 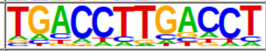 | 1e-28 | -6.592e+01 | 11.73% | 8.06% | 56.2bp (55.3bp) | RARg(NR)/ES-RARg-ChIP-Seq(GSE30538)/Homer(0.965) More Information \| Similar Motifs Found | motif file (matrix) |
| 4 | 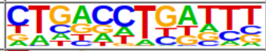 | 1e-28 | -6.539e+01 | 2.56% | 1.04% | 53.8bp (57.6bp) | THRb(NR)/Liver-NR1A2-ChIP-Seq(GSE52613)/Homer(0.711) More Information \| Similar Motifs Found | motif file (matrix) |
| 5 | 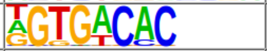 | 1e-26 | -6.201e+01 | 12.58% | 8.88% | 55.1bp (57.6bp) | Tgif2(Homeobox)/mES-Tgif2-ChIP-Seq(GSE55404)/Homer(0.742) More Information \| Similar Motifs Found | motif file (matrix) |
| 6 | 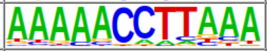 | 1e-17 | -4.067e+01 | 1.61% | 0.66% | 52.8bp (63.8bp) | RUNX3/MA0684.1/Jaspar(0.597) More Information \| Similar Motifs Found | motif file (matrix) |
| 7 | 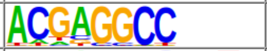 | 1e-17 | -3.977e+01 | 10.51% | 7.77% | 55.3bp (59.5bp) | ZNF711(Zf)/SHSY5Y-ZNF711-ChIP-Seq(GSE20673)/Homer(0.827) More Information \| Similar Motifs Found | motif file (matrix) |
| 8 | 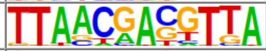 | 1e-15 | -3.476e+01 | 0.23% | 0.02% | 42.9bp (52.7bp) | PH0066.1_Hoxc11/Jaspar(0.717) More Information \| Similar Motifs Found | motif file (matrix) |
| 9 | 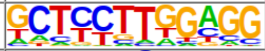 | 1e-13 | -3.206e+01 | 0.21% | 0.01% | 57.0bp (45.8bp) | GLIS3/MA0737.1/Jaspar(0.617) More Information \| Similar Motifs Found | motif file (matrix) |
| 10 | 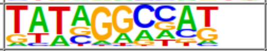 | 1e-13 | -3.124e+01 | 0.69% | 0.20% | 54.0bp (63.4bp) | HOXB13/MA0901.1/Jaspar(0.559) More Information \| Similar Motifs Found | motif file (matrix) |
| 11 | 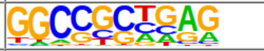 | 1e-13 | -3.096e+01 | 1.89% | 0.95% | 52.6bp (58.8bp) | ZNF711(Zf)/SHSY5Y-ZNF711-ChIP-Seq(GSE20673)/Homer(0.657) More Information \| Similar Motifs Found | motif file (matrix) |
| 12 | 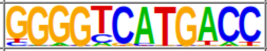 | 1e-13 | -3.063e+01 | 0.84% | 0.28% | 51.7bp (57.6bp) | PB0057.1_Rxra_1/Jaspar(0.740) More Information \| Similar Motifs Found | motif file (matrix) |
| 13 | 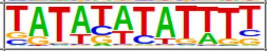 | 1e-12 | -2.979e+01 | 0.28% | 0.03% | 46.9bp (59.7bp) | PB0198.1_Zfp128_2/Jaspar(0.720) More Information \| Similar Motifs Found | motif file (matrix) |
| 14 | 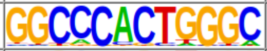 | 1e-12 | -2.835e+01 | 0.36% | 0.06% | 53.1bp (52.5bp) | TFAP2A(var.3)/MA0872.1/Jaspar(0.682) More Information \| Similar Motifs Found | motif file (matrix) |
| 15 | 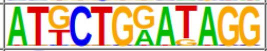 | 1e-12 | -2.773e+01 | 0.12% | 0.00% | 36.7bp (21.7bp) | MAFK/MA0496.1/Jaspar(0.569) More Information \| Similar Motifs Found | motif file (matrix) |
| 16 | 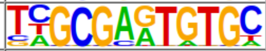 | 1e-12 | -2.773e+01 | 0.12% | 0.00% | 52.9bp (19.4bp) | PB0130.1_Gm397_2/Jaspar(0.750) More Information \| Similar Motifs Found | motif file (matrix) |
| 17 * | 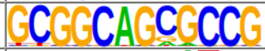 | 1e-11 | -2.749e+01 | 0.22% | 0.02% | 59.4bp (58.6bp) | Sp1(Zf)/Promoter/Homer(0.602) More Information \| Similar Motifs Found | motif file (matrix) |
| 18 * | 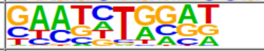 | 1e-11 | -2.547e+01 | 1.70% | 0.89% | 55.7bp (58.0bp) | TEAD4/MA0809.1/Jaspar(0.594) More Information \| Similar Motifs Found | motif file (matrix) |
| 19 * | 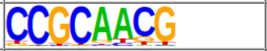 | 1e-10 | -2.522e+01 | 3.01% | 1.89% | 56.1bp (62.0bp) | RUNX2/MA0511.2/Jaspar(0.738) More Information \| Similar Motifs Found | motif file (matrix) |
| 20 * | 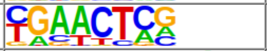 | 1e-10 | -2.500e+01 | 5.17% | 3.66% | 57.1bp (58.7bp) | RXRA::VDR/MA0074.1/Jaspar(0.609) More Information \| Similar Motifs Found | motif file (matrix) |
| [illegible] | 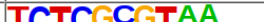 | [illegible] | [illegible] | [illegible] | [illegible] | [illegible] | PH0111.1_Nkx2-2/Jaspar(0.595) | [illegible] |

**Table S5.** GIGGLE output for HaloREVERBα sites

| GSM_ID | Factor | Biosource | GIGGLE_score |
|---|---|---|---|
| GSM890125 | NR1D2 | Epithelium;Liver | 2183.43348 |
| GSM647027 | NCOR1 | Hepatocyte;Liver | 2030.090601 |
| GSM2406335 | HNF4A | Liver | 2011.159414 |
| GSM647029 | NR1D1 | Hepatocyte;Liver | 2000.541779 |
| GSM1659699 | HDAC3 | Liver | 1971.84942 |
| GSM647024 | HDAC3 | Hepatocyte;Liver | 1961.619638 |
| GSM2287957 | HDAC3 | Liver | 1943.174958 |
| GSM1659695 | HDAC3 | Liver | 1931.463445 |
| GSM2406342 | HNF4A | Liver | 1930.922742 |
| GSM2406341 | HNF4A | Liver | 1918.547106 |
| GSM1044697 | HDAC3 | Epithelium;Liver | 1916.908297 |
| GSM2287955 | HDAC3 | Liver | 1913.488117 |
| GSM2287956 | HDAC3 | Liver | 1905.265888 |
| GSM2406329 | HDAC3 | Liver | 1903.926047 |
| GSM2406330 | HDAC3 | Liver | 1896.90569 |
| GSM2406336 | HNF4A | Liver | 1892.608977 |
| GSM2287952 | HDAC3 | Liver | 1886.231319 |
| GSM2287954 | HDAC3 | Liver | 1870.257728 |
| GSM1659696 | HDAC3 | Liver | 1862.933693 |
| GSM2287953 | HDAC3 | Liver | 1857.278636 |
| GSM647028 | NCOR1 | Hepatocyte;Liver | 1849.188389 |
| GSM647023 | HDAC3 | Hepatocyte;Liver | 1838.643677 |
| GSM1163179 | PPARA | Liver | 1819.370416 |
| GSM647022 | HDAC3 | Hepatocyte;Liver | 1819.27416 |
| GSM864672 | PPARA | Hepatocyte;Liver | 1803.517627 |
| GSM2406331 | HDAC3 | Liver | 1790.967104 |
| GSM1163177 | PPARA | Liver | 1789.423476 |
| GSM1163173 | PPARA | Liver | 1771.382774 |
| GSM1163175 | PPARA | Liver | 1756.079456 |
| GSM864674 | RXRA | Hepatocyte;Liver | 1749.982626 |
| GSM864671 | PPARA | Hepatocyte;Liver | 1740.280577 |
| GSM1659697 | HDAC3 | Liver | 1726.916984 |
| GSM864678 | RXRA | Hepatocyte;Liver | 1722.713073 |
| GSM784034 | STAT5B | Hepatocyte;Liver | 1720.547427 |
| GSM864677 | RXRA | Hepatocyte;Liver | 1712.28847 |
| GSM864669 | NR1H2 | Hepatocyte;Liver | 1701.08137 |
| GSM864673 | RXRA | Hepatocyte;Liver | 1700.948838 |
| GSM864676 | RXRA | Hepatocyte;Liver | 1700.912184 |
| GSM2218849 | NR1D1 | Liver | 1700.226489 |
| GSM864668 | NR1H2 | Hepatocyte;Liver | 1700.119511 |
| GSM784035 | STAT5B | Hepatocyte;Liver | 1695.709283 |
| GSM784036 | STAT5B | Hepatocyte;Liver | 1688.784262 |
| GSM784037 | STAT5B | Hepatocyte;Liver | 1684.741875 |
| GSM2218850 | NR1D1 | Liver | 1682.64997 |
| GSM2218847 | NR1D1 | Liver | 1678.782331 |
| GSM2218851 | NR1D1 | Liver | 1675.890775 |
| GSM1163176 | RXRA | Liver | 1672.116631 |

| | | | |
|---|---|---|---|
| GSM1163174 | RXRA | Liver | 1670.005189 |
| GSM864675 | RXRA | Hepatocyte;Liver | 1669.224104 |
| GSM784033 | STAT5B | Hepatocyte;Liver | 1667.554515 |
| GSM1263820 | ESR1 | Epithelium;Liver | 1663.793934 |
| GSM2218846 | NR1D1 | Liver | 1657.242274 |
| GSM840528 | NR1D1 | Epithelium;Liver | 1650.28428 |
| GSM1514929 | PPARA | Epithelium;Liver | 1640.684169 |
| GSM1659689 | NR1D1 | Liver | 1638.885877 |
| GSM784041 | BCL6 | Hepatocyte;Liver | 1637.814977 |
| GSM784040 | BCL6 | Hepatocyte;Liver | 1636.935823 |
| GSM1163172 | RXRA | Liver | 1636.878473 |
| GSM1659693 | RORC | Liver | 1627.561398 |
| GSM784038 | BCL6 | Hepatocyte;Liver | 1615.0622 |
| GSM1463852 | ONECUT1 | Liver | 1612.163861 |
| GSM2699481 | RORA | Liver | 1605.344092 |
| GSM1437734 | RORA | Liver | 1603.032306 |
| GSM717562 | FOXA2 | Hepatocyte;Liver | 1602.869545 |
| GSM634623 | FOXA2 | Hepatocyte;Liver | 1602.869545 |
| GSM1299603 | RXRA | Liver | 1601.210051 |
| GSM1936964 | NR3C1 | L 101;Liver | 1588.070223 |
| GSM1163178 | RXRA | Liver | 1587.187126 |
| GSM2068830 | FOXA2 | Liver | 1585.081425 |
| GSM1936963 | NR3C1 | L 101;Liver | 1582.650403 |
| GSM784039 | BCL6 | Hepatocyte;Liver | 1579.161178 |
| GSM784043 | BCL6 | Hepatocyte;Liver | 1573.085937 |
| GSM1724239 | FOXA2 | 3C10;Liver | 1571.069361 |
| GSM1477607 | FOXA2 | Epithelium;Liver | 1570.141139 |
| GSM982750 | CRY2 | Epithelium;Liver | 1567.460856 |
| GSM784031 | STAT5B | Hepatocyte;Liver | 1561.993883 |
| GSM1138337 | RXRA | Liver | 1559.314539 |
| GSM1299600 | RXRA | Liver | 1559.314539 |
| GSM2218848 | NR1D1 | Liver | 1559.124541 |
| GSM1446062 | NR3C1 | Liver | 1557.779831 |
| GSM1463853 | ONECUT1 | Liver | 1556.601189 |
| GSM1030547 | TBP | 10T1/2;Fibroblast;Embryo | 1555.033111 |
| GSM840529 | NR1D2 | Epithelium;Liver | 1553.425577 |
| GSM864670 | NR1H2 | Hepatocyte;Liver | 1551.128388 |
| GSM1588480 | ARID1A | Hepatocyte;Liver | 1545.633094 |
| GSM1390706 | GTF2E2 | Epithelium;Liver | 1545.0311 |
| GSM784042 | BCL6 | Hepatocyte;Liver | 1541.775505 |
| GSM982748 | CRY2 | Epithelium;Liver | 1537.463406 |
| GSM1236494 | NCOR2 | Epithelium;Liver | 1536.7177 |
| GSM1724234 | FOXA2 | 3C10;Liver | 1534.81178 |
| GSM1854433 | CEBPB | Liver | 1533.734973 |
| GSM1446074 | NR3C1 | Liver | 1533.537235 |
| GSM541305 | RXRA | Epithelium;Liver | 1527.602597 |
| GSM1936965 | NR3C1 | L 101;Liver | 1524.949364 |
| GSM982774 | POLR2A | Epithelium;Liver | 1523.254675 |
| GSM784027 | STAT5B | Hepatocyte;Liver | 1522.240421 |
| GSM982753 | CRY2 | Epithelium;Liver | 1522.13382 |
| GSM1211424 | ESR1 | Liver | 1521.456415 |

| | | | |
|---|---|---|---|
| GSM982773 | POLR2A | Epithelium;Liver | 1520.085638 |
| GSM1390699 | TBP | Epithelium;Liver | 1519.671267 |
| GSM1659687 | NR1D1 | Liver | 1518.292957 |
| GSM982781 | POLR2A | Epithelium;Liver | 1518.29246 |
| GSM1659686 | NR1D1 | Liver | 1512.922211 |
| GSM1384739 | FOXA2 | Liver | 1512.532722 |
| GSM1854437 | CEBPB | Liver | 1508.235425 |
| GSM1390703 | GTF2B | Epithelium;Liver | 1508.046852 |
| GSM1446072 | NR3C1 | Liver | 1507.353969 |
| GSM1446064 | NR3C1 | Liver | 1503.865292 |
| GSM982779 | POLR2A | Epithelium;Liver | 1501.814302 |
| GSM541292 | RXRA | Epithelium;Liver | 1500.507728 |
| GSM2055885 | CEBPB | Liver | 1500.449589 |
| GSM982731 | PER1 | Epithelium;Liver | 1500.037154 |
| GSM2406344 | HNF4A | Liver | 1498.9951 |
| GSM878679 | CUX2 | Epithelium;Liver | 1498.164972 |
| GSM967657 | TBP | TSC;Trophoblast Stem Cell;Embryo | 1497.126492 |
| GSM1390704 | GTF2B | Epithelium;Liver | 1496.263974 |
| GSM1446071 | NR3C1 | Liver | 1495.827147 |
| GSM1854435 | CEBPB | Liver | 1495.568214 |
| GSM982777 | POLR2A | Epithelium;Liver | 1494.83651 |
| GSM1390705 | GTF2E2 | Epithelium;Liver | 1494.415547 |
| GSM2102917 | POLR2A | Mammary Gland | 1494.279826 |
| GSM1390700 | TBP | Epithelium;Liver | 1493.85364 |
| GSM864689 | POLR2A | Hepatocyte;Liver | 1493.831068 |
| GSM1106101 | CDK9 | Embryonic Fibroblast;Embryo | 1492.865879 |
| GSM982780 | POLR2A | Epithelium;Liver | 1492.32652 |
| GSM1037673 | POLR2A | Epithelium;Liver | 1492.044067 |
| GSM2406343 | HNF4A | Liver | 1490.244493 |
| GSM423364 | POLR2A | Neural Progenitor Cell;Embryo | 1489.940084 |
| GSM1446063 | NR3C1 | Liver | 1489.734067 |
| GSM541304 | RXRA | Epithelium;Liver | 1488.487382 |
| GSM982782 | POLR2A | Epithelium;Liver | 1486.362001 |
| GSM1854432 | CEBPB | Liver | 1483.989521 |
| GSM2406338 | HNF4A | Liver | 1482.937646 |
| GSM982778 | POLR2A | Epithelium;Liver | 1482.877728 |
| GSM1463855 | ONECUT1 | Liver | 1482.850586 |
| GSM864683 | POLR2A | Hepatocyte;Liver | 1482.140241 |
| GSM2055884 | FOXA3 | Liver | 1480.690358 |
| GSM1301670 | CLOCK | Liver | 1480.625377 |
| GSM541301 | RXRA | Epithelium;Liver | 1480.562139 |
| GSM2055886 | CEBPB | Liver | 1479.700154 |
| GSM982746 | CRY1 | Epithelium;Liver | 1477.846077 |
| GSM1463857 | ONECUT1 | Liver | 1477.613594 |
| GSM1236495 | NCOR2 | Epithelium;Liver | 1477.134236 |
| GSM541295 | RXRA | Epithelium;Liver | 1475.121913 |
| GSM982751 | CRY2 | Epithelium;Liver | 1474.830513 |
| GSM863769 | TCF4 | Hepatocyte | 1474.039259 |
| GSM982737 | PER2 | Epithelium;Liver | 1473.402112 |
| GSM1437733 | NFIL3 | Liver | 1473.137772 |
| GSM982736 | PER2 | Epithelium;Liver | 1470.586395 |

| | | | |
|---|---|---|---|
| GSM1446065 | NR3C1 | Liver | 1469.596156 |
| GSM864687 | POLR2A | Hepatocyte;Liver | 1467.725323 |
| GSM2790896 | BRDT | Skeletal Muscle | 1467.185397 |
| GSM982752 | CRY2 | Epithelium;Liver | 1467.166719 |
| GSM1463859 | ONECUT1 | Liver | 1465.149893 |
| GSM918738 | POLR2A | Epithelium;Liver | 1465.105278 |
| GSM2055887 | HNF4A | Liver | 1463.847654 |
| GSM2633466 | BRD4 | Preadipocyte | 1463.550631 |
| GSM874951 | POLR2B | Epithelium;Liver | 1458.862009 |
| GSM1382346 | POLR2A | Fibroblast;Embryo | 1457.108117 |
| GSM2406332 | PROX1 | Liver | 1456.758334 |
| GSM2100638 | TBP | Testis | 1456.009931 |
| GSM982738 | PER2 | Epithelium;Liver | 1455.213386 |
| GSM541300 | RXRA | Epithelium;Liver | 1455.208271 |
| GSM1463856 | ONECUT1 | Liver | 1455.196457 |
| GSM982743 | CRY1 | Epithelium;Liver | 1454.047397 |
| GSM2100637 | TBP | Testis | 1453.380479 |
| GSM982745 | CRY1 | Epithelium;Liver | 1452.967436 |
| GSM982757 | CREBBP | Epithelium;Liver | 1452.016604 |
| GSM542588 | POLR2A | Hepatocyte;Liver | 1450.611062 |
| GSM2470637 | BRD4 | AT-3 | 1449.816267 |
| GSM784030 | STAT5B | Hepatocyte;Liver | 1449.721241 |
| GSM1514931 | PPARA | Epithelium;Liver | 1449.423749 |
| GSM1947436 | POLR2A | B Lymphocyte | 1449.317336 |
| GSM1199137 | POLR2A | 3T3-L1;Preadipocyte;Adipose | 1449.289952 |
| GSM1865397 | POLR2A | C2C12;Myoblast;Muscle | 1448.72629 |
| GSM1272601 | THRB | Liver | 1448.541893 |
| GSM1635081 | POLR2A | lens | 1448.179111 |
| GSM2406337 | HNF4A | Liver | 1447.978376 |
| GSM917525 | POLR2A | Embryonic Fibroblast;Embryo | 1447.97538 |
| GSM1947442 | POLR2A | B Lymphocyte | 1446.780764 |
| GSM1106084 | POLR2A | Embryonic Fibroblast;Embryo | 1446.568615 |
| GSM1111748 | NR3C1 | Liver | 1446.196358 |
| GSM982688 | ARNTL | Epithelium;Liver | 1446.120337 |
| GSM982758 | CREBBP | Epithelium;Liver | 1445.992735 |
| GSM2087162 | ESR2 | Epididymal White Adipose Tissue | 1445.821914 |
| GSM851319 | POLR2A | Epithelium;Placenta | 1445.594093 |
| GSM1106087 | POLR2A | Embryonic Fibroblast;Embryo | 1443.871784 |
| GSM864681 | POLR2A | Hepatocyte;Liver | 1443.77893 |
| GSM2220070 | POLR2A | Liver | 1443.450411 |
| GSM2065678 | POLR2A | Epithelium;Intestine | 1442.964878 |
| GSM864679 | POLR2A | Hepatocyte;Liver | 1442.593231 |
| GSM1267724 | POLR2A | Epithelium;Forelimb | 1442.457422 |
| GSM864685 | POLR2A | Hepatocyte;Liver | 1441.109221 |
| GSM864688 | POLR2A | Hepatocyte;Liver | 1441.06736 |
| GSM917531 | POLR2A | Embryonic Fibroblast;Embryo | 1440.515769 |
| GSM1087778 | ARNTL | Liver | 1440.391539 |
| GSM2790892 | BRD4 | Skeletal Muscle | 1439.679395 |
| GSM541306 | POLR2A | Epithelium;Liver | 1439.577082 |
| GSM1122543 | POLR2A | Epithelium;Liver | 1439.397792 |
| GSM1199136 | POLR2A | 3T3-L1;Preadipocyte;Adipose | 1439.118223 |

**Table S6.** Reactome pathway enrichment of differentially expressed genes in liver of *Reverbα*$^{Flox2\text{-}6}$*Alb*$^{CreERT2}$ mice (20 terms with smallest Padj values shown).

| ID | Description | GeneRatio | BgRatio | pvalue | p.adjust | qvalue | Count |
|---|---|---|---|---|---|---|---|
| R-MMU-425366 | Transport of bile salts and organic acids, metal ions and amine compounds | 8/116 | 76/8828 | 6.25E-06 | 0.00238 | 0.0023 | 8 |
| R-MMU-425407 | SLC-mediated transmembrane transport | 11/116 | 226/8828 | 0.00018 | 0.034251 | 0.0334 | 11 |
| R-MMU-383280 | Nuclear Receptor transcription pathway | 5/116 | 50/8828 | 0.000475 | 0.060317 | 0.0588 | 5 |
| R-MMU-204998 | Cell death signalling via NRAGE, NRIF and NADE | 5/116 | 68/8828 | 0.001937 | 0.184534 | 0.18 | 5 |
| R-MMU-425410 | Metal ion SLC transporters | 3/116 | 21/8828 | 0.002474 | 0.18852 | 0.1839 | 3 |
| R-MMU-193648 | NRAGE signals death through JNK | 4/116 | 49/8828 | 0.003803 | 0.231702 | 0.226 | 4 |
| R-MMU-193704 | p75 NTR receptor-mediated signalling | 5/116 | 83/8828 | 0.004619 | 0.231702 | 0.226 | 5 |
| R-MMU-170834 | Signaling by TGF-beta Receptor Complex | 4/116 | 54/8828 | 0.005399 | 0.231702 | 0.226 | 4 |
| R-MMU-73887 | Death Receptor Signalling | 6/116 | 124/8828 | 0.005745 | 0.231702 | 0.226 | 6 |
| R-MMU-2022090 | Assembly of collagen fibrils and other multimeric structures | 4/116 | 57/8828 | 0.006544 | 0.231702 | 0.226 | 4 |
| R-MMU-194840 | Rho GTPase cycle | 6/116 | 128/8828 | 0.00669 | 0.231702 | 0.226 | 6 |
| R-MMU-1251985 | Nuclear signaling by ERBB4 | 2/116 | 12/8828 | 0.010367 | 0.290013 | 0.2828 | 2 |
| R-MMU-1475029 | Reversible hydration of carbon dioxide | 2/116 | 12/8828 | 0.010367 | 0.290013 | 0.2828 | 2 |
| R-MMU-1236394 | Signaling by ERBB4 | 3/116 | 35/8828 | 0.010657 | 0.290013 | 0.2828 | 3 |
| R-MMU-200425 | Import of palmitoyl-CoA into the mitochondrial matrix | 2/116 | 14/8828 | 0.014051 | 0.310599 | 0.3029 | 2 |
| R-MMU-2173791 | TGF-beta receptor signaling in EMT (epithelial to mesenchymal transition) | 2/116 | 14/8828 | 0.014051 | 0.310599 | 0.3029 | 2 |
| R-MMU-5633008 | TP53 Regulates Transcription of Cell Death Genes | 2/116 | 14/8828 | 0.014051 | 0.310599 | 0.3029 | 2 |
| R-MMU-416482 | G alpha (12/13) signalling events | 4/116 | 72/8828 | 0.014674 | 0.310599 | 0.3029 | 4 |
| R-MMU-194068 | Bile acid and bile salt metabolism | 3/116 | 44/8828 | 0.019807 | 0.395401 | 0.3856 | 3 |
| R-MMU-1474290 | Collagen formation | 4/116 | 81/8828 | 0.021702 | 0.395401 | 0.3856 | 4 |

**Table S7.** Reactome pathway enrichment of differentially expressed genes in liver of *Reverbα*$^{-/-}$ mice (20 terms with smallest Padj values shown).

| ID | Description | GeneRatio | BgRatio | pvalue | p.adjust | qvalue | Count |
|---|---|---|---|---|---|---|---|
| R-MMU-1428517 | The citric acid (TCA) cycle and respiratory electron transport | 73/1649 | 133/8828 | 5.40E-21 | 5.73E-18 | 4.87E-18 | 73 |
| R-MMU-1430728 | Metabolism | 459/1649 | 1730/8828 | 1.07E-19 | 5.70E-17 | 4.85E-17 | 459 |
| R-MMU-163200 | Respiratory electron transport, ATP synthesis by chemiosmotic coupling, and heat production by uncoupling proteins. | 51/1649 | 82/8828 | 3.05E-18 | 1.08E-15 | 9.18E-16 | 51 |
| R-MMU-611105 | Respiratory electron transport | 38/1649 | 59/8828 | 1.21E-14 | 3.20E-12 | 2.72E-12 | 38 |
| R-MMU-6799198 | Complex I biogenesis | 35/1649 | 54/8828 | 1.03E-13 | 2.18E-11 | 1.86E-11 | 35 |
| R-MMU-77289 | Mitochondrial Fatty Acid Beta-Oxidation | 24/1649 | 36/8828 | 3.43E-10 | 6.07E-08 | 5.16E-08 | 24 |
| R-MMU-72766 | Translation | 78/1649 | 223/8828 | 3.95E-09 | 5.99E-07 | 5.09E-07 | 78 |
| R-MMU-8953854 | Metabolism of RNA | 150/1649 | 551/8828 | 2.43E-07 | 3.22E-05 | 2.74E-05 | 150 |
| R-MMU-5419276 | Mitochondrial translation termination | 36/1649 | 85/8828 | 3.57E-07 | 4.21E-05 | 3.58E-05 | 36 |
| R-MMU-5368287 | Mitochondrial translation | 36/1649 | 86/8828 | 5.05E-07 | 5.36E-05 | 4.56E-05 | 36 |
| R-MMU-5389840 | Mitochondrial translation elongation | 35/1649 | 83/8828 | 5.88E-07 | 5.67E-05 | 4.82E-05 | 35 |
| R-MMU-392499 | Metabolism of proteins | 369/1649 | 1607/8828 | 1.06E-06 | 9.38E-05 | 7.98E-05 | 369 |
| R-MMU-1592230 | Mitochondrial biogenesis | 16/1649 | 26/8828 | 1.63E-06 | 0.0001328 | 0.0001129 | 16 |
| R-MMU-163210 | Formation of ATP by chemiosmotic coupling | 12/1649 | 18/8828 | 1.05E-05 | 0.0007448 | 0.0006333 | 12 |
| R-MMU-8949613 | Cristae formation | 12/1649 | 18/8828 | 1.05E-05 | 0.0007448 | 0.0006333 | 12 |
| R-MMU-72203 | Processing of Capped Intron-Containing Pre-mRNA | 69/1649 | 228/8828 | 1.27E-05 | 0.000845 | 0.0007185 | 69 |
| R-MMU-9609507 | Protein localization | 38/1649 | 106/8828 | 2.06E-05 | 0.0012839 | 0.0010916 | 38 |
| R-MMU-74160 | Gene expression (Transcription) | 237/1649 | 1015/8828 | 4.38E-05 | 0.0024087 | 0.002048 | 237 |
| R-MMU-8978868 | Fatty acid metabolism | 53/1649 | 169/8828 | 4.40E-05 | 0.0024087 | 0.002048 | 53 |
| R-MMU-196780 | Biotin transport and metabolism | 8/1649 | Oct-28 | 4.58E-05 | 0.0024087 | 0.002048 | 8 |

**Table S8.** Primer sequences for qPCR

| Gene | Forward Primer (5'-3') | Reverse Primer (5'-3') |
|---|---|---|
| *Aacs* | CGACAGAGTCGCCCTTTACG | TGTTGGGTAGATAGCCGACCA |
| *Actb* | GGCTGTATTCCCCTCCATCG | CCAGTTGGTAACAATGCCATGT |
| *Aldh1a1* | ATACTTGTCGGATTTAGGAGGCT | GGGCCTATCTTCCAAATGAACA |
| *Aldh1a7* | ACTTGGAAGTTAGGCCCTGC | TGTGAAGGACACTTTGTCGATG |
| *Bmal1* | GTCGAATGATTGCCGAGGAA | GGGAGGCGTACTTGTGATGTTC |
| *Cox6a1* | TCAACGTGTTCCTCAAGTCGC | AGGGTATGGTTACCGTCTCCC |
| *Cry1* | TCGCCGGCTCTTCCAA | TCAAGACACTGAAGCAAAAATCG |
| *Hprt* | GTTGGATACAGGCCAGACTTTGTTG | GATTCAACTTGCGCTCATCTTAGGC |
| *Irs2* | ACCGACTTGGTCAGCGAAG | CACGAGCCCGTAGTTGTCAT |
| *Lhpp* | GACATCTCCGGGGTGCTATG | CTTTCAGCGGGGACTGTTTCA |
| *Ndufa8* | GGAGCTGCCAACTCTGGAAG | CCAGCGGCACAGCATAAAC |
| *Ndufs7* | GTTCATCAGAGTGTAGCCACTG | CAGGCCGAAGGTCATAGGC |
| *Nfil3* | GCTCTTTTGTGGACGAGCAT | ACCGAGGACACCTCTGACAC |
| *Npas2* | CAGCTTGAACCCAAAGGAAT | CCATTACAGGAGGGGCTAGG |
| *Pcsk4* | CTCAACATCCTAAAGGCTTGGAA | GGGTGGTCTTTCTCAATGCC |
| *Per1* | TGAAGCAAGACCGGGAGAG | CACACACGCCATCACATCAA |
| *Per2* | GCCTTCAGACTCATGATGACAGA | TTTGTGTGCCTCAGCTTTGG |
| *Plin2* | AAGAGGCCAAACAAAAGAGCCAGGAGACCA | ACCCTGAATTTTCTGGTTGGCACTGTGCAT |
| *Plin5* | TGTCCAGTGCTTACAACTCGG | CAGGGCACAGGTAGTCACAC |
| *Ppib* | GGAGATGGCACAGGAGGAAA | CCGTAGTGCTTCAGTTTGAAGTTCT |
| *Rev-erbα* | GTCTCTCCGTTGGCATGTCT | CCAAGTTCATGGCGCTCT |
| *Rev-erbβ* | CAGGAGGTGTGATTGCCTACA | GGACGAGGACTGGAAGCTAAT |
| *Scd1* | CGCTGGTGCCCTGGTACTGC | CAGCCAGGTGGCGTTGAGCA |
| *Uck1* | ACCGCTAGTGGCAAGTCAAC | AGAACCTTGTAGAAGCAGTCCT |
| *Uqcr11* | CACATGCGTAGTGCTCCAGG | CGGCTGTGGGAATCCAGTTT |

**Table S9.** Published datasets used

| Dataset | Source | Identifier |
|---|---|---|
| Genome Reference Consortium Mouse Build 38 (mm10) | http://genome.ucsc.edu/cgi-bin/hgGateway?db=mm10 | GCA_000001635.2 |
| GENCODE Release M16 mouse genome annotation | https://www.gencodegenes.org/mouse/release_M16.html | GENCODE M16 |
| Liver REVERBα ZT8 ChIP-seq raw data | Cho et al., 2012 | GEO: GSE34020; SRR385650 |
| Liver ZT8 input raw data | Cho et al., 2012 | GEO: GSE34020; SRR385652 |
| Liver REVERBα ZT10 WT ChIP-seq raw data (two replicates) | Kim et al., 2018 | GEO: GSE104129; SRR6059484, SRR6059485 |
| Liver REVERBα ZT10 REVERBα KO ChIP-seq raw data (two replicates) | Kim et al., 2018 | GEO: GSE104129; SRR6059486, SRR6059487 |
| Liver HDAC3 ZT10 ChIP-seq raw data (three replicates) | Feng et al., 2011 | GEO: GSE26345; SRR351386, SRR351387, SRR351388 |
| Liver NCOR ZT10 ChIP-seq raw data | Feng et al., 2011 | GEO: GSE26345; SRR351391 |
| Liver ZT10 input raw data | Feng et al., 2011 | GEO: GSE26345; SRR351397 |
| Liver ZT6 DNase-seq raw data | Sobel et al., 2017 | GEO: GSE60430; SRR1551954 |
| Liver ZT10 DNase-seq raw data | Sobel et al., 2017 | GEO: GSE60430; SRR1551955 |
| Circadian gene rhythmicity analysis results (mouse liver) | Koike et al., 2012 | Tables S6, S7 from that paper |
| Mouse liver TAD boundaries (BED file) | Kim et al., 2018 | GEO: GSE104129; GSE104129_MainTads_List_mm9.bed.gz |
| Mouse liver subTAD boundaries (BED file) | Kim et al., 2018 | GEO: GSE104129; GSE104129_SubTads-List_mm9.bed.gz |
| Liver ZT10 RNA-seq raw counts data | Guan et al., 2020 | GEO: GSE143524; GSE143524_Ad_lib_feeding_counts.txt.gz |
